# Temporal relationship between atherogenic dyslipidemia and inflammation and their joint cumulative effect on type 2 diabetes onset: a longitudinal cohort study

**DOI:** 10.1186/s12916-023-02729-6

**Published:** 2023-01-24

**Authors:** Yulong Lan, Guanzhi Chen, Dan Wu, Xiong Ding, Zegui Huang, Xianxuan Wang, Lois Balmer, Xingang Li, Manshu Song, Wei Wang, Shouling Wu, Youren Chen

**Affiliations:** 1 Department of Cardiology, Second Affiliated Hospital of Shantou University Medical College, 69 Dongxia North Rd, Shantou, 515041 China; 2 Centre for Precision Health, Edith Cowan University, Building 21/270 Joondalup Drive, Perth, WA 6027 Australia; 3 China Medical University, Shenyang, 110122 China; 4 Department of Pediatrics, Second Affiliated Hospital of Shantou University Medical College, Shantou, 515041 China; 5 School of Public Health, Wuhan University, Wuhan, 430072 China; 6 Beijing Key Laboratory of Clinical Epidemiology, School of Public Health, Capital Medical University, Beijing, 100069 China; 7 School of Public Health, Shandong First Medical University & Shandong Academy of Medical Sciences, Tai’an, 271099 China; 8 Department of Cardiology, Kailuan General Hospital, 57 Xinhua East Rd, Tangshan, 063000 China

**Keywords:** Inflammation, Dyslipidemia, Type 2 diabetes, Temporal relationship Longitudinal study

## Abstract

**Background:**

Concurrent atherogenic dyslipidemia and elevated inflammation are commonly observed in overt hyperglycemia and have long been proposed to contribute to diabetogenesis. However, the temporal relationship between them and the effect of their cumulative co-exposure on future incident type 2 diabetes (T2D) remains unclear.

**Methods:**

Longitudinal analysis of data on 52,224 participants from a real-world, prospective cohort study (Kailuan Study) was performed to address the temporal relationship between high-sensitivity C-reactive protein (hsCRP) and the atherogenic index of plasma (AIP, calculated as triglyceride/high-density lipoprotein) in an approximately 4-year exposure period (2006/2007 to 2010/2011). After excluding 8824 participants with known diabetes, 43,360 nondiabetic participants were included for further analysis of the T2D outcome. Cox regression models were used to examine the adjusted hazard ratios (aHRs) upon the cumulative hsCRP (CumCRP) and AIP (CumAIP) in the exposure period.

**Results:**

In temporal analysis, the adjusted standardized correlation coefficient (β1) of hsCRP_2006/2007 and AIP_2010/2011 was 0.0740 (95% CI, 0.0659 to 0.0820; *P* < 0.001), whereas the standardized correlation coefficient (β2) of AIP_2006/2007 and hsCRP_2010/2011 was − 0.0293 (95% CI, − 0.0385 to − 0.0201; *P* < 0.001), which was significantly less than β1 (*P* < 0.001). During a median follow-up of 7.9 years, 5,118 T2D cases occurred. Isolated exposure to CumAIP or CumCRP was dose-dependently associated with T2D risks, independent of traditional risk factors. Significant interactions were observed between the median CumAIP (− 0.0701) and CumCRP thresholds (1, 3 mg/L) (*P* = 0.0308). Compared to CumAIP < − 0.0701 and CumCRP < 1 mg/L, those in the same CumAIP stratum but with increasing CumCRP levels had an approximately 1.5-fold higher T2D risk; those in higher CumAIP stratum had significantly higher aHRs (95% CIs): 1.64 (1.45–1.86), 1.87 (1.68–2.09), and 2.04 (1.81–2.30), respectively, in the CumCRP < 1, 1 ≤ CumCRP < 3, CumCRP ≥ 3 mg/L strata. Additionally, the T2D risks in the co-exposure were more prominent in nonhypertensive, nondyslipidemic, nonprediabetic, or female participants.

**Conclusions:**

These findings suggest a stronger association between elevated hsCRP and future AIP changes than vice versa and highlight the urgent need for combined assessment and management of chronic inflammation and atherogenic dyslipidemia in primary prevention, particularly for those with subclinical risks of T2D.

**Supplementary Information:**

The online version contains supplementary material available at 10.1186/s12916-023-02729-6.

## Background

The increased prevalence of type 2 diabetes (T2D) is a burgeoning health threat worldwide [1]. T2D enhances the risk of cardiovascular disease (CVD) [2], kidney dysfunction [3], and chronic disease mortality [4]. Moreover, even with early active multifactorial interventions among diabetic groups, only limited improvements were reported, as the incidence of the first CVD events and mortality were not significantly improved [5]. Indeed, shifting the clinical priority from progressive and expensive diabetes management to early proactive strategies for T2D prevention and remission has been substantially emphasized [6].

Atherogenic dyslipidemia, including changes in lipid panels routinely determined in clinical practice and multiple modified lipoproteins [7], is a common hallmark in the diabetic or diabetes-prone milieu, characterized by high triglyceride (TG) and low high-density lipoprotein cholesterol (HDL-C) levels [8]. In addition, systemic inflammation is often observed in T2D concurrent with metabolic dyslipidemia [9]. It is well known that inflammation plays a key role in diabetogenesis, inducing islet beta-cell inflammation [10, 11], impairing beta-cell functions [11], and enhancing insulin resistance (IR) [12, 13]. Notably, both atherogenic lipid changes and low-grade inflammation have been identified as biologically entangled processes [14]. The atherogenic dyslipidemia complex, mostly derived from obesity, enhances a low-grade inflammatory circumstance by the lipotoxic effect and persistent leakage of cytokines [15]. In turn, inflammation greatly mediates lipid metabolism, modifying the constitution and fraction of lipid profiles and deteriorating IR [16, 17]. A genetic study indicated a polygenic overlap between C-reactive protein and plasma lipids (e.g., TGs, HDL) and signaled a need for a combination of variants involved in inflammation or lipid metabolism in the risk assessment of cardiometabolic diseases [18]. The preponderance of evidence suggests an urgency for aiding in translation of the biologically intertwined relationship into epidemiological practice, thereby informing the primary prevention strategies against T2D among the general population. Furthermore, despite the long-standing observation of concomitant changes in atherogenic dyslipidemia and elevated inflammation, limited studies have examined the temporal relationship between them.

To fill this knowledge gap, we therefore conducted a longitudinal study based on the data from a prospective cohort (the Kailuan Study) to examine the risk of incident T2D, with a cumulative atherogenic index of plasma (AIP, calculated by TG/HDL) and high-sensitivity CRP (hsCRP) within an approximately 4-year period predating the follow-up as the exposure. Together, we conducted a path analysis to address the temporal relationship between hsCRP and AIP changes in the exposure period.

## Methods

### Study participants

Initiating in 2006, the Kailuan Study (trial registration number: ChiCTR–TNC–11001489) was an ongoing, prospective, cohort study conducted in Tangshan, China. The following health surveys were issued every 2 years, with a total of seven surveys through to December 31, 2020. Details of the study design have been provided previously [19, 20]. Each participant provided written informed consent before enrollment. The current subanalysis of the Kailuan Study was approved by the Kailuan General Hospital Ethics Committee, China (2006–05) and the Human Research Ethics Committee of Edith Cowan University (2021–03159–BALMER).

**Fig. 1:**
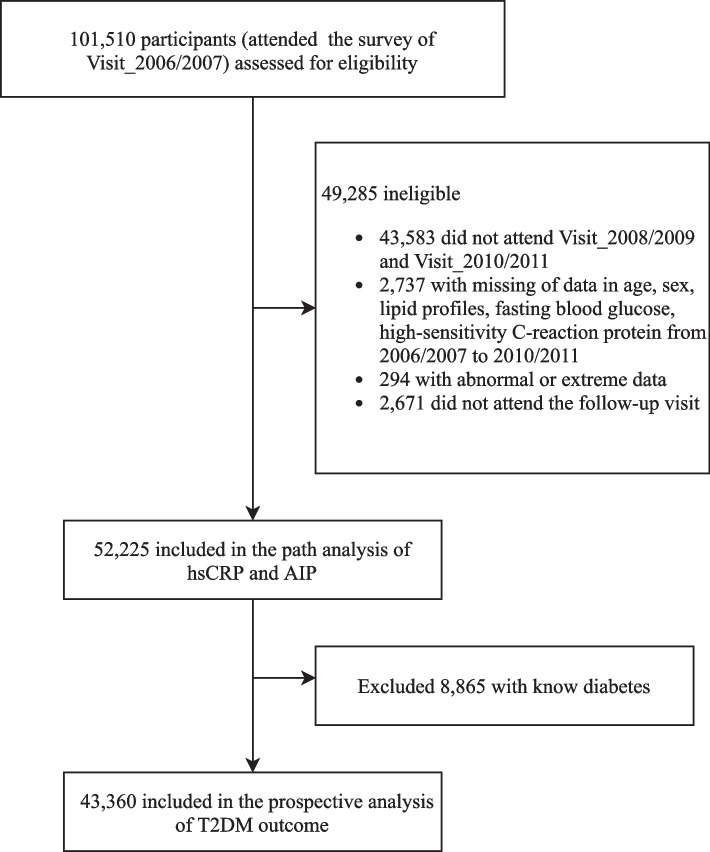
Flowchart of the study participants

Fig. 1 displays the flowchart of participant enrollment in this current study. Among 101,510 participants who attended the first survey in 2006/2007, we excluded a total of 49,285 participants, including those who did not attend the following two health surveys (*n* = 43,583); those who had incomplete information on sex, age, or abnormal lipid profiles and high-sensitivity C-reactive protein (hsCRP) data (*n* = 3031); and those who missed any of the follow-up visits (*n* = 2671), leaving 52,225 participants in the path analysis. We further excluded those with preexisting diabetes before the commencement of follow-up (2010/2011) (*n* = 8865) for the survival analysis. Finally, a total of 43,360 participants were included in the prospective analysis of the T2D outcome. The number of participants and participations in the four follow-up visits and participants who attended each follow-up visit are reported in Additional file 1: Table S1.

### Ascertainment of outcome

The primary outcome of this study was the incidence of T2D (International Classification of Diseases–10 [ICD–10]: E11). T2D was diagnosed as either fasting blood glucose (FBG) ≥ 7.0 mmol/L, a self-reported history of T2D diagnosis, or self-reported medication uses of oral antihyperglycemic agents [21]. The date of T2D onset was defined as the first of the available follow-up examinations at which a participant met the diagnostic criteria. Participants contributed their follow-up time until the occurrence of T2D or death or the last available follow-up visit.

### Exposure assessment

**Fig. 2:**
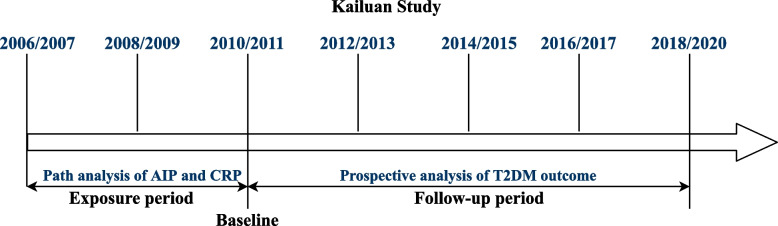
Strategies and design of the current study. The health examinations in the Kailuan Study were provided around every 2 years, except for the current last visit, with a time span of approximately 3 years owing to the influence of the COVID-19 pandemic. For the current study, the path analysis addressing the temporal relationship between AIP and hsCRP was based on data measured in 2006/2007 and 2010/2011. For the survival analysis of T2DM outcome, the cumulative exposure period was from 2006/2007 to 2010/2011. At the end of Visit_2010/2011, the nondiabetic participants were followed up biannually through December 31, 2020. Baseline characteristics were based on the information in Visit_2010/2011

A brief summary of the study design and procedures of this cohort is provided in Fig. 2. The cumulative exposure was attained in a median 3.95 [interquartile range (IQR): 3.73–4.29] years of period predating the follow-up. Chronic inflammation was measured by cumulative hsCRP (CumCRP), calculated as (hsCRP_2006/2007 + hsCRP_2008/2009)/2 × (visit 1 − 2) + (hsCRP_2008/2009 + hsCRP_2010/2011)/2 × (visit 2 − 3), where hsCRP_2006/2007, hsCRP_2008/2009, and hsCRP_2009/2010 were the hsCRP levels measured at 2006/2007, 2008/2009, and 2010/2011 physical examinations, respectively, and visit 1 − 2 and visit 2 − 3 indicated the time intervals between the two health surveys. Cumulative AIP (CumAIP) and other lipid characteristics were calculated using the same algorithm. As no clear threshold for cumulative hsCRP currently exists and the high intraindividual variability over time supports repeat measurements for ensuring a stable assessment, we used the suggested clinical cutoffs (< 1, 1 to 3, ≥ 3 mg/L) for transient hsCRP in the Asian population [22, 23] as the CumCRP thresholds. The comparison of CumCRP and CumAIP to the mean value of each transient measure in the exposure period is displayed in Additional file 1: Table S2.

### Covariates

As described previously [20], the data on sociodemographic characteristics, anthropometric measurements, biochemistry parameters for evaluating systemic health (lipid profiles, hsCRP, creatine, FBG), and lifestyle factors (alcohol consumption, smoking) as well as past medical and medication history (diabetes, CVD, hypertension, fatty liver, and current treatments including antihypertensives, antidiabetic, and lipid-lowering agents) were collected via standardized questionnaires. Anthropometric measurements of height, weight, and blood pressure were conducted by trained physicians following a standard protocol. Blood pressure was categorized as normal blood pressure, grade I hypertension, grade II hypertension, and grade III hypertension [24]. Fatty liver was routinely assessed by abdominal ultrasonography by experienced radiologists using a high-resolution B-mode topographical ultrasound system with a 3.5-MHz probe (ACUSON X300, Siemens, Germany). The severity of fatty liver was categorized as mild, moderate, or severe. The estimated glomerular filtration rate (eGFR) was calculated from creatinine levels following the Chronic Kidney Disease Epidemiology Collaboration formula [25]. Body mass index (BMI) was calculated as weight (kg) divided by height squared (m^2^). Current smokers were defined as smoking at least one cigarette/day on average in a recent year. Drinking status was defined according to average alcohol consumption in the past year.

### Statistical analysis

Multiple imputation by chained equation techniques was performed for missing values in potential covariables (> 98% complete). We examined the temporal relationship between hsCRP and AIP in the exposure period (median period of 3.95 years) with a typical cross-lagged panel design, as demonstrated in Additional file 1: Fig. S1. This design measured the path coefficient (β1) of hsCRP_2006/2007 on AIP_2010/2011 and the path coefficient (β2) of AIP_2006/2007 on hsCRP_2010/2011 simultaneously, adjusting for the auto-regressive effects. A significant path coefficient (β1 or β2) suggests directionality, and a significant difference between β1 and β2 provides stronger evidence for the directional path between the two variables measured over time. Statistical differences in β1 and β2 were examined using a *t* test. In this analysis, hsCRP was log-transformed, and then both log (hsCRP) and AIP were standardized to means as 0 and standard deviation (SD) as 1. The multivariable-adjusted models were as follows: model 1 was adjusted for age, sex, lipid-lowering drugs, antihypertensive drug use, smoking habits, alcohol consumption, BMI, FBG, systolic blood pressure (SBP), eGFR, and LDL-C measured in 2006/2007, and model 2 was further adjusted for the time interval between examinations (years).

For baseline descriptions of the nondiabetic participants in the prospective analysis of T2D risks, the mean and SD, median and IQR, or frequency and percentage (%) were used, as appropriate. Differences in the baseline characteristics across the six CumAIP-by-CumCRP strata were compared by ANOVA (for continuous normally distributed variables), Kruskal–Wallis test (for continuous skew-distributed variables) and *χ*^2^ test (for categorical variables). Because of skewed distribution, CumCRP, hsCRP, HDL-C, and TG were log-transformed as continuous variables, and eGFR was divided into 4 categories according to clinical cut-points: ≥ 90, 60~90, 30~60, and < 30, in the model analyses.

Incidence rates of T2D were calculated as per 1000 person-years. The association between CumAIP and T2D incidence, with or without stratifying by CumCRP thresholds, was examined utilizing multivariable Cox proportional hazards regression models. The association between CumCRP alone and incident T2D was investigated using weighted Cox proportional hazards models because of the violation of the proportional hazards’ assumption. Hazard ratios (HRs) with 95% confidence intervals (CIs) were calculated. *P* values for trend and risks per SD increment of CumAIP or log (CumCRP) were calculated. The multiplicative interaction (INTm) between CumAIP and CumCRP was tested with the likelihood ratio test. The joint effect of CumAIP and CumCRP on the risk of developing T2D was further investigated using multivariable-adjusted models: model 1 was adjusted for age, sex, education, smoking, alcohol consumption, physical activities, family history of diabetes, and BMI; model 2 was further adjusted for FBG, eGFR, total cholesterol (TC), blood pressure, antihypertensives (yes or no), and lipid-lowering drugs (yes or no); and model 3 was additionally adjusted for fatty liver degree. To compare the overall survival of each risk group of CumAIP-by-CumCRP strata based on follow-up intervals, the Kaplan–Meier plots were generated, and the log-rank test was conducted. Further INTm analyses were performed between joint cumulative exposure and baseline sex, overweight, hypertensive, dyslipidemia, or impaired fasting glucose (IFG) status. Stratified analyses among these covariates were performed according to the identified interactions. In addition, sensitivity analyses were performed to assess the robustness and consistency of the findings. Firstly, we excluded participants with baseline CVD to minimize the influence of potential confounds on the lipid and inflammation biomarkers and T2D risk. Secondly, we excluded study endpoints that occurred within the first follow-up visit to address the potential reverse causation. Thirdly, we excluded those with any hsCRP level ≥ 10 mg/L during the exposure period. Fourthly, we performed an analysis with the raw datasets (without imputation for missing values). Fifthly, since the data with the study endpoint of T2D were interval-censored survival datasets, we additionally performed the analyses with the SAS ICPHREG procedure to fit a proportional hazards model on the interval-censored survival data. Sixthly, we further performed the survival analyses by adjusting for the time-varying covariates in the follow-up period. At each date of an event (T2D), the model used the covariates present at the visit just before the event.

All statistical analyses were conducted in the SAS software (version 9.4; SAS Institute, Cary, NC) with the SAS Proc Calis procedure for cross-lagged analysis. A two-sided *P* value < 0.05 was considered statistically significant, except for a *P* value < 0.1 in the interaction testing.

## Results

### Temporal relationship between hsCRP and AIP

**Fig. 3:**
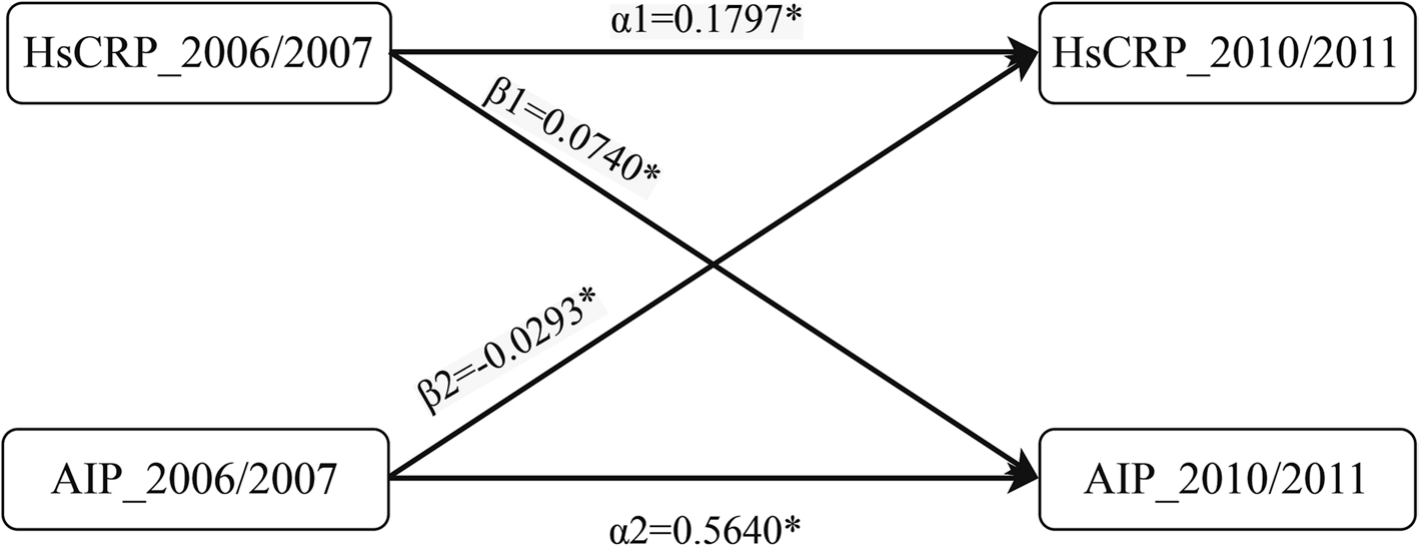
Cross-lagged standard regression coefficient of hsCRP and AIP (*n* = 52,225). *P < 0.001. The cross-lagged model was adjusted for age, sex, education, smoking status, drinking status, physical activities, family history of diabetes, BMI, FBG, SBP, TC, eGFR (categorical), antihypertensives, lipid-lowering drugs measured in 2006/2007, and time intervals. *Abbreviations*: AIP, atherogenic index of plasma; BMI, body mass index; eGFR, estimated glomerular filtration rate; FBG, fasting blood glucose; HsCRP, high-sensitivity C-reactive protein; SBP, systolic blood pressure; TC, total cholesterol

The results of the temporal analysis of hsCRP and AIP are displayed in Fig. 3 and Additional file 1: Table S3. In the fully adjusted model, the standardized correlation coefficient (β1) of hsCRP_2006/2007 and AIP_2010/2011 was 0.0740 (95% CI, 0.0659 to 0.0820; *P* < 0.001), whereas the standardized correlation coefficient (β2) of AIP_2006/2007 and hsCRP_2010/2011 was − 0.0293 (95% CI, − 0.0385 to − 0.0201; *P* < 0.001), which was significantly less than β1 (*P* < 0.001). This indicated a bidirectional relationship between AIP and hsCRP, and the effect of hsCRP on future AIP change was more prominent than vice versa. All correlation coefficients between log (hsCRP) and AIP measured in 2006/2007 and 2010/2011 were statistically significant (Additional file 1: Table S4).

### Baseline characteristics of 43,360 nondiabetic participants

Baseline characteristics were determined according to the information on 43,360 nondiabetic individuals at the start of the follow-up (Table 1). There was a male skewness (32,618 [75.2%]) in the study participants, with a mean (SD) age of 52.1 (11.8) at the onset of follow-up. Participants with higher CumCRP and CumAIP levels had higher BMI, blood pressure (SBP and DBP), FBG, CumTC and baseline TC, CumTG, and baseline TG and higher prevalence of hypertension, moderate and severe fatty liver, as well as more use of antihypertensive and lipid-lowering agents, whereas they had lower eGFR and HDL levels. Additionally, those in the higher CumCRP and CumAIP strata were more likely to be males, current drinkers, current smokers, and physically inactive. Furthermore, age increased with increasing CumCRP levels. However, for each CumCRP stratum, those with elevated CumAIP levels tended to be younger.

**Table 1:** Baseline characteristics of the study participants

Characteristics	Total	CumAI***P*** < − 0.0701 and CumCRP < 1 mg/L	CumAI***P*** < − 0.0701 and 1 ≤ CumCRP < 3 mg/L	CumAI***P*** < − 0.0701 and CumCRP ≥ 3 mg/L	CumAI***P*** ≥ − 0.0701 and CumCRP < 1 mg/L	CumAI***P*** ≥ − 0.0701 and 1 ≤ CumCRP < 3 mg/L	CumAI***P*** ≥ − 0.0701 and CumCRP ≥ 3 mg/L	***P*** value
**Cumulative characteristics of lipid profiles and hsCRP**								
CumHDL-C, median (IQR), mmol/L	1.5 (1.3, 1.8)	1.7 (1.5, 1.9)	1.7 (1.5, 1.9)	1.7 (1.5, 1.9)	1.4 (1.2, 1.6)	1.4 (1.2, 1.6)	1.4 (1.2, 1.6)	< 0.0001
CumLDL-C, mean (SD), mmol/L	2.6 (0.7)	2.5 (0.7)	2.6 (0.7)	2.5 (0.9)	2.6 (0.6)	2.7 (0.7)	2.6 (0.8)	< 0.0001
CumTG, median (IQR), mmol/L	1.3 (1.0, 1.9)	0.9 (0.7, 1.1)	1.0 (0.8, 1.2)	1.0 (0.8, 1.2)	1.8 (1.4, 2.4)	1.9 (1.5, 2.6)	2.0 (1.5, 2.7)	< 0.0001
CumTC, mean (SD), mmol/L	5.1 (1.0)	4.9 (0.9)	5.1 (1.0)	5.1 (1.0)	5.1 (1.0)	5.2 (1.0)	5.4 (1.1)	< 0.0001
CumAIP, mean (SD)	− 0.06 (0.27)	− 0.24 (0.17)	− 0.21 (0.15)	− 0.22 (0.16)	0.18 (0.17)	0.20 (0.18)	0.22 (0.19)	< 0.0001
CumCRP, median (IQR), mg/L	1.5 (0.8, 3.0)	0.6 (0.4, 0.8)	1.7 (1.3, 2.2)	5.1 (3.8, 7.6)	0.7 (0.5, 0.8)	1.7 (1.3, 2.2)	4.9 (3.8, 7.1)	< 0.0001
**Baseline characteristics**								
Age, mean (SD), years	52.1 (11.8)	50.2 (11.7)	52.4 (12.0)	56.0 (12.2)	50.0 (11.0)	51.5 (11.5)	54.4 (11.7)	< 0.0001
Male (%)	32,618 (75.2)	5353 (66.2)	6173 (71.9)	3620 (72.3)	4969 (83.9)	8113 (81.4)	4390 (75.8)	< 0.0001
AIP, mean (SD)	− 0.06 (0.31)	− 0.24 (0.23)	− 0.20 (0.22)	− 0.18 (0.22)	0.17 (0.26)	0.20 (0.26)	0.22 (0.26)	< 0.0001
BMI, mean (SD), kg/m^2^	25.0 (3.3)	23.3 (2.9)	24.3 (3.2)	24.8 (3.4)	25.0 (2.9)	26.0 (3.1)	26.7 (3.4)	< 0.0001
SBP, mean (SD), mmHg	129.5 (18.6)	124.1 (17.9)	128.5 (18.8)	131.0 (19.5)	128.4 (17.4)	131.8 (18.0)	134.1 (18.8)	< 0.0001
DBP, median (IQR), mmHg	80.7 (79.3, 90.0)	80.0 (72.0, 88.3)	80.0 (78.0, 90.0)	80.0 (79.3, 90.0)	82.0 (80.0, 90.0)	84.7 (80.0, 90.0)	85.0 (80.0, 90.7)	< 0.0001
FBG, mean (SD), mmol/L	5.2 (0.6)	5.2 (0.5)	5.2 (0.6)	5.2 (0.6)	5.3 (0.6)	5.3 (0.6)	5.3 (0.6)	< 0.0001
HDL-C, median (IQR), mmol/L	1.5 (1.3, 1.8)	1.7 (1.4, 2.1)	1.7 (1.4, 2.0)	1.6 (1.4, 1.9)	1.4 (1.2, 1.6)	1.4 (1.2, 1.6)	1.3 (1.1, 1.6)	< 0.0001
LDL-C, mean (SD), mmol/L	2.6 (0.8)	2.5 (0.7)	2.7 (0.7)	2.2 (0.9)	2.6 (0.7)	2.8 (0.7)	2.5 (1.0)	< 0.0001
TC, mean (SD), mmol/L	5.0 (1.0)	4.9 (1.0)	4.9 (0.9)	4.9 (0.9)	5.0 (0.9)	5.1 (1.0)	5.1 (1.1)	< 0.0001
TG, median (IQR), mmol/L	1.3 (0.9–1.8)	0.9 (0.7, 1.2)	1.0 (0.7–1.2)	1.0 (0.7–1.3)	1.6 (1.2–2.3)	1.8 (1.3–2.5)	1.8 (1.4–2.6)	< 0.0001
eGFR, median (IQR), mL/min/1.73 m^2^	82.1 (59.7, 97.0)	89.4 (66.3, 102.0)	85.4 (60.4, 98.3)	78.1 (58.7, 93.1)	74.3 (57.5, 96.1)	78.9 (58.4, 95.8)	73.7 (58.0, 93.0)	< 0.0001
HsCRP, median (IQR), mg/L	1.0 (0.5, 2.4)	0.5 (0.3, 0.9)	1.3 (0.7, 2.3)	3.1 (1.1, 6.4)	0.5 (0.1, 0.9)	1.2 (0.6, 2.2)	3.1 (1.2, 6.1)	< 0.0001
Current drinker (%)	15,201 (35.1)	2761 (34.1)	2822 (32.9)	1324 (26.4)	2534 (42.8)	3925 (39.4)	1835 (31.7)	< 0.0001
Current smokers (%)	14,717 (33.9)	2444 (30.2)	2735 (31.9)	1423 (28.4)	2296 (38.8)	3831 (38.4)	1988 (34.3)	< 0.0001
Family history of diabetes	2300 (5.3)	448 (5.5)	414 (4.8)	215 (4.3)	334 (5.6)	570 (5.7)	319 (5.5)	0.0011
Education (%)								< 0.0001
Less than senior high school	32,892 (75.9)	5745 (71.0)	6496 (75.7)	4060 (81.1)	4419 (74.6)	7517 (75.4)	4655 (80.4)	
Senior high school and above	10,468 (24.1)	2341 (29.0)	2089 (24.3)	949 (18.9)	1506 (25.4)	2449 (24.6)	1134 (19.6)	
Physical activities (%)								< 0.0001
Low	14,568 (33.6)	2971 (36.7)	2711 (31.6)	1671 (33.4)	2048 (34.6)	3224 (32.3)	1943 (33.6)	
Moderate	22,710 (52.4)	3943 (48.8)	4538 (52.9)	2745 (54.8)	3057 (51.6)	5327 (53.5)	3100 (53.5)	
High	6082 (14.0)	1172 (14.5)	1336 (15.6)	593 (11.8)	820 (13.8)	1415 (14.2)	746 (12.9)	
Hypertension (%)	20,881 (48.2)	2762 (34.2)	3751 (43.7)	2533 (50.6)	2825 (47.7)	5495 (55.1)	3515 (60.7)	< 0.0001
Dyslipidemia (%)	11,821 (27.3)	960 (11.9)	1046 (12.2)	695 (13.9)	2201 (37.1)	4223 (42.4)	2696 (46.6)	< 0.0001
Moderate and severe fatty liver (%)	5785 (13.3%)	278 (3.4%)	598 (6.9%)	475 (9.5%)	754 (12.7%)	2169 (21.8%)	1611 (26.1%)	< 0.0001
Anti-hypertensives (%)	2305 (5.3)	218 (2.7)	360 (4.2)	277 (5.5)	308 (5.2)	640 (6.4)	502 (8.7)	< 0.0001
Statins	252 (0.6)	26 (0.3)	31 (0.4)	20 (0.4)	38 (0.6)	69 (0.7)	68 (1.2)	< 0.0001
Fibrate	95 (0.2)	1 (0.0)	7 (0.1)	6 (0.1)	18 (0.3)	30 (0.3)	33 (0.6)	< 0.0001

*Abbreviations*: *BMI*, body mass index; *CumAIP*, cumulative atherogenic index of plasma; *CumCRP*, cumulative high-sensitivity C-reactive protein; *CumHDL-C*, cumulative high-density lipoprotein cholesterol; *CumLDL-C*, cumulative low-density lipoprotein cholesterol; *CumTC*, cumulative total cholesterol; *CumTG*, cumulative triglyceride; *DBP*, diastolic blood pressure; *eGFR*, estimated glomerular filtration rate; *FBG*, fasting blood glucose; *HDL-C*, high-density lipoprotein cholesterol; *HsCRP*, high-sensitivity C-reactive protein; *LDL-C*, low-density lipoprotein cholesterol; *SBP*, systolic blood pressure; *TC*, total cholesterol; *TG*, triglyceride

### Prospective study of the T2D risks upon CumAIP and CumCRP

During a median of 7.9 (IQR: 5.7–8.9) years of follow-up, 5118 incident T2D cases were documented among 43,360 participants. Both isolated CumAIP and CumCRP were positively associated with the risk of T2D onset. The aHRs (95% CIs) were 1.26 (1.14–1.39), 1.55 (1.41–1.70), and 2.03 (1.85–2.22) respectively in CumAIP quartiles 2, 3, and 4 vs. quartile 1 after adjustment for traditional risk factors. Additional adjustment for fatty liver degree further attenuated the risks, leaving aHRs (95% CIs) of 1.23 (1.11–1.35), 1.44 (1.31–1.58), and 1.75 (1.60–1.92) in CumAIP quartiles 2, 3, 4 vs. quartile 1 (*P* for trend < 0.001; Table 2). In terms of CumCRP, the incidence rates of T2D and T2D risks increased with increasing CumCRP levels, with an average HR (95% CI) of 1.12 (1.09–1.16) per SD increase in logCumCRP (0.4279) (*P*-trend < 0.001; Additional file 1: Table S5).

**Table 2:** CumAIP-associated T2D risks in the entire cohort and across the cumulative CRP thresholds (1, 3 mg/L)

	CumAIP, HRs (95% CIs)				Per SD^a^	***P*** for trend
	Quartile1	Quartile 2	Quartile 3	Quartile 4		
Entire cohort						
Event/total	684/10, 840	1023/10, 840	1395/10, 840	2016/10, 840		
Incident rate	8.6	13.37	18.6	27.32		
Model 1	Reference	1.37 (1.24, 1.51)	1.76 (1.60, 1.93)	2.40 (2.20, 2.63)	1.36 (1.32, 1.39)	< 0.0001
Model 2	Reference	1.26 (1.14, 1.39)	1.55 (1.41, 1.70)	2.03 (1.85, 2.22)	1.28 (1.24, 1.31)	< 0.0001
Model 3	Reference	1.23 (1.11, 1.35)	1.44 (1.31, 1.58)	1.75 (1.60, 1.92)	1.20 (1.17, 1.24)	< 0.0001
CumCRP < 1 mg/L						
Event/total	191/4462	238/3624	321/3287	388/2638		
Incident rate	5.76	9.2	13.88	21.35		
Model 1	Reference	1.38 (1.14, 1.67)	1.92 (1.59, 2.30)	2.78 (2.31, 3.33)	1.47 (1.39, 1.56)	< 0.0001
Model 2	Reference	1.27 (1.05, 1.54)	1.59 (1.32, 1.91)	2.28 (1.90, 2.75)	1.36 (1, 29, 1.45)	< 0.0001
Model 3	Reference	1.24 (1.02, 1.50)	1.48 (1.23, 1.79)	1.99 (1.65, 2.40)	1.28 (1.20, 1.36)	< 0.0001
1 ≤ CumCRP < 3 mg/L						
Event/total	304/3952	485/4633	658/4932	954/5034		
Incident rate	10.55	14.83	19.31	27.85		
Model 1	Reference	1.26 (1.10, 1.46)	1.55 (1.35, 1.77)	2.13 (1.86, 2.44)	1.31 (1.26, 1.37)	< 0.0001
Model 2	Reference	1.15 (0.99, 1.32)	1.37 (1.20, 1.58)	1.81 (1.58, 2.07)	1.25 (1.20, 2.30)	< 0.0001
Model 3	Reference	1.14 (0.98, 1.31)	1.31 (1.14, 1.50)	1.62 (1.41, 1.85)	1.19 (1.14, 1.24)	< 0.0001
CumCRP ≥ 3 mg/L						
Event/total	189/2426	300/2583	416/2621	674/3168		
Incident rate	10.79	16.74	23.38	31.55		
Model 1	Reference	1.41 (1.18, 1.70)	1.84 (1.54, 2.18)	2.31 (1.96, 2.73)	1.30 (1.24, 1.36)	< 0.0001
Model 2	Reference	1.34 (1.12, 1.61)	1.68 (1.41, 2.01)	2.00 (1.69, 2.37)	1.23 (1.17, 1.29)	< 0.0001
Model 3	Reference	1.30 (1.08, 1.56)	1.58 (1.32, 1.88)	1.73 (1.46, 2.06)	1.16 (1.10, 1.22)	< 0.0001
*P* for INTm: CumAIP quartile × CumCRP thresholds (1, 3 mg/L) = 0.0308; CumAIP quartiles × logCumCRP = 0.0286						

Quartile 1: CumAIP < − 0.2387; quartile 2: − 0.2387 ≤ CumAIP < − 0.0701; quartile 3: − 0.0701 ≤ CumAIP < 0.0989; quartile 4: CumAIP > 0.0989

Model 1: adjusted for baseline age, sex, education, smoking, drinking status, physical activities, family history of diabetes, BMI; model 2: model 1 + FBG, TC, blood pressure (categorical), eGFR (categorical), antihypertensives, lipid-lowering drugs, logBasCRP (continuous, only in the entire cohort); model 3: model 2 + fatty liver (categorical)

*Abbreviations*: *CumAIP*, cumulative atherogenic index of plasma; *CumCRP*, cumulative high-sensitivity C-reactive protein; *BMI*, body mass index; *FBG*, fasting blood glucose; *TC*, total cholesterol; *eGFR*, estimated glomerular filtration rate; *BasCRP*: baseline high-sensitivity C-reactive protein; *INTm*, multiplicative interaction

^a^Per SD, hazard ratio for per SD (0.2658) change in CumAIP

There was a significant interaction between CumAIP quartiles and CumCRP tested at clinical thresholds (1, 3 mg/L) (*P*–INTm: 0.0308; and *P*–INTm = 0.0286 when CumCRP was tested as a log-transformed continuous variable) (Table 2). The CumAIP-associated T2D risks were significant in all CumCRP strata; however, they varied greatly across different CumCRP strata. The risk in the top CumAIP quartile was markedly higher in the CumCRP < 1 mg/L stratum (HR: 1.99, 95% CI: 1.65–2.40) and lower in the 1 ≤ CumCRP < 3 mg/L stratum (HR: 1.60, 95% CI: 1.33–1.92), while it was moderate in the CumCRP ≥ 3 mg/L stratum (HR: 1.73, 95% CI: 1.46–1.06).

**Fig. 4:**
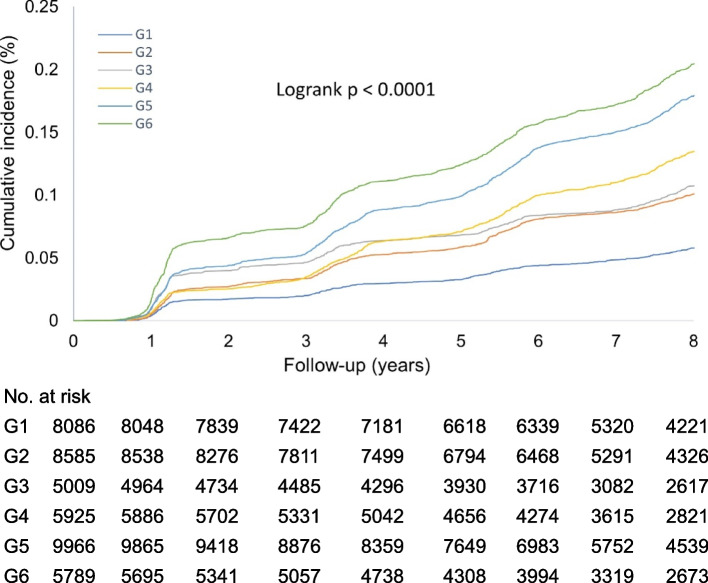
Kaplan–Meier curves of the cumulative incidence of T2D over a mean of 7.9 years follow-up across CumAIP-CumCRP subgroups. G1: CumAIP < − 0.0701 and CumCRP < 1 mg/L; G2: CumAIP < − 0.0701 and 1 ≤ CumCRP < 3 mg/L; G3: CumAIP < − 0.0701 and CumCRP ≥ 3 mg/L; G4: CumAIP ≥ − 0.0701 and CumCRP < 1 mg/L; G5: CumAIP ≥ − 0.0701 and 1 ≤ CumCRP < 3 mg/L; G6: CumAIP ≥ − 0.0701 and CumCRP ≥ 3 mg/L. G1 was used as the reference group

Further subgroup analysis of the joint exposure was conducted by stratifying with medium CumAIP and CumCRP thresholds (1, 3 mg/L; Table 3). Compared to CumAIP < − 0.0701 and CumCRP < 1 mg/L, those in the same CumAIP stratum but with increasing CumCRP levels had an approximately 1.5-fold higher risk of incident T2D; those in higher CumAIP stratum (CumAIP ≥ − 0.0701) had significantly higher risks in all CumCRP levels, with aHRs (95% CIs) of 1.64 (1.45–1.86), 1.87 (1.68–2.09), and 2.04 (1.81–2.30) respectively in CumCRP < 1 mg/L, 1 ≤ CumCRP < 3 mg/L, CumCRP ≥ 3 strata. Figure 4 displays the Kaplan–Meier curves of the cumulative incidence of T2D in the overall study participants. We also tested the T2D risks with different references (Additional file 1: Table S6). All results collectively demonstrated that in the same CumAIP stratum, lower CumCRP markedly decreased the risks of incident T2D; for those with the same CumCRP level, low CumAIP enhanced a significantly protective effect against T2D onset. For example, in those with CumCRP < 1 mg/L, lower CumAIP levels significantly decreased the T2D risks (HR: 0.61, 95% CI: 0.54–0.69) compared to those in the higher CumAIP stratum.

**Table 3:** Type 2 diabetes risk upon co-exposure stratified by CumCRP thresholds (1, 3 mg/L) and CumAIP (median)

	Combination of CumCRP and CumAIP, HRs (95% CIs)					
	G1	G2	G3	G4	G5	G6
Event/total	429/8086	789/8585	489/5009	709/5932	1612/9966	1090/5789
Incidence rate^a^	7.27	12.82	13.80	17.17	23.59	27.84
Model (unadjusted)	Reference	1.77 (1.58, 1.99)	1.90 (1.67, 2.16)	2.35 (2.08, 2.65)	3.25 (2.92, 3.61)	3.84 (3.43, 4.29)
Model 1	Reference	1.54 (1.37, 1.73)	1.47 (1.29, 1.68)	1.97 (1.75, 2.23)	2.41 (2.16, 2.69)	2.55 (2.27, 2.86)
Model 2	Reference	1.49 (1.32, 1.68)	1.52 (1.33, 1.73)	1.75 (1.55, 1.98)	2.12 (1.90, 2.36)	2.38 (2.11, 2.67)
Model 3	Reference	1.58 (1.40, 1.78)	1.44 (1.27, 1.65)	1.64 (1.45, 1.86)	1.87 (1.68, 2.09)	2.04 (1.81, 2.30)
*P* for interaction: CumAIP median × logCumCRP = 0.0113; CumAIP median × CumCRP cut-points (1, 3 mg/L) = 0.0369						

G1: CumAIP < − 0.0701 and CumCRP < 1 mg/L; G2: CumAIP < − 0.0701 and 1 ≤ CumCRP < 3 mg/L; G3: CumAIP < − 0.0701 and CumCRP ≥ 3 mg/L; G4: CumAIP ≥ − 0.0701 and CumCRP < 1 mg/L; G5: CumAIP ≥ − 0.0701 and 1 ≤ CumCRP < 3 mg/L; G6: CumAIP ≥ − 0.0701 and CumCRP ≥ 3 mg/L

Model 1: adjusted for age, sex, education, smoking, drinking status, physical activities, family history of diabetes, and BMI

Model 2: model 1 + FBG, TC, blood pressure (categorical), eGFR (categorical), antihypertensives, and lipid-lowering drugs

Model 3: model 2 + fatty liver (categorical)

*Abbreviations*: *CumAIP*, cumulative atherogenic index of plasma; *CumCRP*, cumulative high-sensitivity C-reactive protein; *HR*, hazard ratio; *BMI*, body mass index; *FBG*, fasting blood glucose; *TC*, total cholesterol; *eGFR*, estimated glomerular filtration rate; *INTm*, multiplicative interaction

^a^The incidence rate is per 1000 person-years

Similar significant results were found in the sensitivity analyses when excluding participants with baseline CVD or T2D cases that occurred within the first follow-up visit or on the raw data without imputation; however, decreased risks were observed with the exclusion of those with any hsCRP value ≥ 10 mg/L during the exposure period (Additional file 1: Table S7). Additionally, the repeated survival analysis with the ICPHREG procedure to fit the interval-censored data yield similar results to the main results (Additional file 1: Tables S8-S10). Furthermore, the associations between the study exposures and incident T2D in the time-varying analyses were similarly robust to those in the fixed-covariates Cox models with only baseline values, albeit somewhat stronger (Additional file 1: Tables S11-S13).

In the stratified analyses, a significant interaction was found between co-exposure and sex (*P*–INTm = 0.0034; Additional file 1: Table S14); female participants had significantly higher risks of T2D when suffering from dyslipidemia and aberrant inflammation, with an HR (95% CI) of 2.38 (1.86–3.03) in females vs. 1.95 (1.70–2.24) in males. The presence of IFG in the exposure period (*P*–INTm: 0.0365; Additional file 1: Table S15), baseline hypertension (*P*–INTm: 0.0005; Additional file 1: Table S16), or dyslipidemia (*P*–INTm: 0.0619; Additional file 1: Table S17) further attenuated the risks of T2D compared to absence. However, no statistically significant interaction between baseline overweight status and co-exposure was observed to be associated with T2D onset.

We additionally assessed the risks of T2D upon co-exposure to inflammation and dyslipidemia when measuring in a transient pattern (baseline hsCRP and AIP). Likewise, significant interactions between baseline hsCRP thresholds (1, 3 mg/L) and AIP were observed (Additional file 1: Tables S18, S19). The aHRs (95% CIs) of incident T2D were significant in the higher AIP stratum and increased with increasing hsCRP levels (1.37 [1.25–1.50], 1.21 [1.10–1.33], 1.35 [1.22–1.50] respectively for hsCRP < 1 mg/L, 1 ≤ hsCRP < 3 mg/L, hsCRP ≥ 3 mg/L), whereas they only remained significant in the lower AIP strata when concomitant with a hsCRP level ≥ 3 mg/L (1.13 [1.01–1.27]).

## Discussion

For the first time, among 52,225 general participants, our findings supported a bidirectional relationship between hsCRP and atherogenic dyslipidemia in a temporal analysis, and the effect of inflammation on future AIP change was more robust than vice versa. Additionally, among 43,360 participants without preexisting diabetes, we observed that both isolated cumulative AIP and hsCRP were significantly associated with T2D risk, independent of age, sex, medication use, and baseline measures of FBG, TC, hsCRP, blood pressure, and fatty liver degree. Importantly, our findings showed a significant interaction between chronic atherogenic dyslipidemia and inflammation associated with developing T2D. In particular, the diabetic risks upon joint exposures were significantly higher in female, nonhypertensive, nondyslipidemic, and nonprediabetic participants.

Dual changes in inflammation and dyslipidemia have been suggested to persist and predate T2D onset, and both are intertwined biological processes [14, 15]. Although atherogenic dyslipidemia concomitant with elevated hsCRP levels has long been observed [26, 27], the temporal relationship between them remains unexplored. Our path analysis supported a bidirectional correlation between them among the general population. Although obesity-derived dyslipidemia is well known to enhance a proinflammatory milieu through continuous activation of inflammatory cytokines in circulation and the lipotoxic effect of free fatty acids as well as ceramide biosynthesis [15], our findings evidenced a stronger effect of elevated hsCRP on future AIP. Indeed, inflammation and its cytokine-mediated changes in lipase activity (e.g., incretins, lipoprotein lipase, and cholesteryl ester transfer protein activity) potentially drive the alteration of atherogenic dyslipidemia [16, 17]. Furthermore, mounting evidence suggests that altered atherogenic lipids are secondary to IR [28, 29]. A study consisting of 509 nondiabetic adults demonstrated that elevated hsCRP is associated with future IR by using the same cross-lagged analysis [30], to an extent, supporting our findings. Interestingly, a negative association between AIP and future hsCRP was observed in the current study. We proposed that the great involvement of multifactorial influences on hsCRP, for example, age-specific metabolic and inflammatory profiles, may underlie this observation [31]. The cross-sectional data at baseline showed that age persistently increased with increasing CumCRP levels but decreased with CumAIP levels in each CumCRP stratum. As aging is a major source of inflammation, it is possible that age mediated the negative association between AIP and hsCRP. Collectively, our findings provide novel perspectives of the temporal relationship between elevated inflammation and atherogenic dyslipidemia and warrant further explorations to unravel the underlying causative processes between them.

In addition, our study demonstrated that both cumulative AIP and hsCRP were independently associated with an increased risk of incident T2D. Irrespective of the debatable role of transient hsCRP measures for the risk assessment of T2D onset [32, 33], our study showed that cumulative hsCRP was stably associated with future T2D risks, independent of traditional risk factors. Converging studies have demonstrated that atherogenic lipid changes and inflammation are entangled processes underlying the foundation of IR and insulin deficiency. This current study, for the first time, provided an epidemiological link between CumCRP and CumAIP, supporting a need for a combined assessment of the intertwined process (es) from the epidemiological landscape. The difference in T2D risks conferred by dyslipidemia across different levels of CumCRP is of interest. Those with persistently low hsCRP levels (CumCRP < 1 mg/L) had the highest risk, and those with significantly elevated hsCRP levels (CumCRP > 3 mg/L) had moderate risk, while those with modest hsCRP levels (1 ≤ CumCRP < 3 mg/L) had the lowest risk. Several interpretations may underlie this finding. Aside from the abovementioned obesity [14, 34], diverse factors, such as smoking, chronic stress, hypertension, and aging, enhance the low-grade inflammatory milieu [31]. As such, the attenuation of T2D risks conferred by chronic dyslipidemia in the 1 ≤ CumCRP < 3 mg/L stratum is likely because other risk factors majorly contribute to diabetogenesis in this setting. However, in the CumCRP ≥ 3 mg/L stratum, the influence of high-grade inflammation (hsCRP ≥ 10 mg/L [23]) was potentially included. It has been reported that certain acute infections enhance high-grade inflammation and boost the progression to T2D onset [35, 36]. Therefore, the inclusion of high-grade inflammation and potential acute infection processes in the CumCRP ≥ 3 mg/L stratum would enhance higher T2D risks in dyslipidemia. This is supported by the decreased risks of T2D in the CumCRP-by-CumAIP strata in the sensitivity analyses with exclusion of any hsCRP ≥ 10 mg/L during the exposure period.

These findings have substantial clinical implications. Clinical assessment of the risks of T2D is guided by temporary hsCRP and TG/HDL measures, whereas our findings suggested that incorporating cumulative AIP and inflammatory burden into clinical practice may further refine T2D risk assessment and help inform strategies for primary prevention. The significant interactions between inflammation and atherogenic dyslipidemia in developing T2D and between their co-exposures and hypertensive, dyslipidemia, and IFG statuses as well as sex effect signal a need for a combined assessment of chronic inflammatory burden and dyslipidemia and strict risk stratification in precise individualized intervention. More ideal cardiovascular health metrics confer a lower risk of T2D. In accordance with the significant interaction observed between chronic inflammation and dyslipidemia, measures potentiating dual-target benefits warrant further attention. Intensive lifestyle interventions, e.g., calorie restriction [37], higher intake of dietary fiber [38], habitual physical exercise [39], and smoking cessation [40], are well-established approaches to achieve improvements in both dyslipidemia and inflammation.

This current study has several strengths. Firstly, it was conducted with data from a well-established prospective cohort, where a rigorous quality assurance program was performed to ensure data validity and reliability. Secondly, this study extends previous reports by using different metrics—cumulative hsCRP and AIP in an approximately 4-year exposure period, thus overcoming the weakness of the surveys and risk prediction tools which are mostly cross-sectional in primary or secondary prevention, as the prolonged exposure is the true cause in diabetogenesis. Thirdly, ongoing controversies regarding the intraindividual variability over time [22] strengthen the need for repeated measurements of hsCRP in a longitudinal design. Our longitudinal investigation of data from a large study population ensured stable and reliable results reflecting the true relationship among the investigated risk factors. Fourthly, the unique temporal analysis applied in this current study contributes to addressing the concerning science question regarding the entangled relationship between inflammation and atherogenic dyslipidemia among the general population.

The limitations of the current study also need to be addressed. This community-based cohort study primarily consisted of Han Chinese individuals, potentially limiting generalizability to the whole population. Furthermore, we had no available insulin data for the assessment of IR, yet an additional adjustment for fatty liver may compensate for this limitation because fatty liver is a well-known risk factor for T2D with mechanistic involvement of systemic inflammation, hepatic IR, oxidative stress, and lipid metabolism. In addition, we failed to distinguish T2D and type 1 diabetes (T1D). Nevertheless, the misclassification of T1D was minimal, given that T2D predominates 95% of all diabetes, and the average age among the study participants at follow-up (52.1 years) is greater than the generally observed T1D onset age. Furthermore, given that there is a lack of a clear threshold for cumulative hsCRP levels, the use of the suggested transient hsCRP cutoff values for CumCRP may lead to bias. However, the strict measurement taking changes over time into consideration has potentiated CumCRP as a more reliable parameter to reflect the inflammatory burden.

## Conclusions

Our findings suggest a stronger influence of elevated hsCRP on future atherogenic lipid changes than vice versa. The cumulative inflammatory burden modifies the risk of T2D conferred by atherogenic dyslipidemia. Dual assessment and management of chronic inflammation and atherogenic dyslipidemia are instrumental for T2D prevention, especially for nonhypertensive, nondyslipidemic, nonprediabetic, or female individuals.

## Supplementary Information

**Additional file 1: Table S1.** Number of participants and participations in the follow-up visits. **Table S2.** Value of CumAIP, mean AIP, CumCRP, and mean hsCRP during the exposure period. **Table S3.** Multivariable adjusted cross-lagged standard regression coefficient of hsCRP and AIP (n=52,225). **Table S4.** Pearson correlation coefficients between log-transformed hsCRP and HOMA-IR at baseline and follow-up in the total cohort and subgroups, adjusted for covariates. **Table S5.** CumCRP-associated risk for incident type 2 diabetes. **Table S6.** Type 2 diabetes risk with co-exposure stratified by CumCRP thresholds (1, 3 mg/L) and CumAIP (median) relative to different references. **Table S7.** Sensitivity analyses of incidence of type 2 diabetes with co-exposure stratified by CumCRP thresholds (1, 3 mg/L) and CumAIP (median). **Table S8.** Sensitivity analysis of CumAIP-associated type 2 diabetes risks in the entire cohort and across the cumulative CRP thresholds (1, 3 mg/L) using the ICPHREG procedure. **Table S9.** Sensitivity analysis of CumCRP-associated T2D risks in the entire cohort using the ICPHREG procedure. **Table S10.** Sensitivity analysis of co-exposure–associated type 2 diabetes risks in the entire cohort using the ICPHREG procedure. **Table S11.** CumAIP–associated type 2 diabetes risks in the entire cohort and across the cumulative CRP thresholds (1, 3 mg/L) with adjustment for time-varying covariates. **Table S12.** CumCRP-associated type 2 diabetes risks with adjustment for time-varying covariates. **Table S13.** The co-exposure-associated type 2 diabetes risks with adjustment for time-varying covariates. **Table S14.** Association between co-exposure to CumAIP and CumCRP and type 2 diabetes incidence stratified by sex. **Table S15.** Association between co-exposure to CumAIP and CumCRP and type 2 diabetes incidence stratified by IFG status in exposure period. **Table S16.** Association between co-exposure to CumAIP and CumCRP and type 2 diabetes incidence stratified by baseline blood pressure. **Table S17.** Association between co-exposure to CumAIP and CumCRP and type 2 diabetes incidence stratified by dyslipidemia status. **Table S18.** BasAIP-associated T2D in the entire cohort and across the baseline hsCRP thresholds (1, 3 mg/L). **Table S19.** Type 2 diabetes risk on co-exposure stratified by BasCRP thresholds (1, 3 mg/L) and BasAIP (median). **Fig. S1.** Cross-lagged analysis design panel.

## Data Availability

The datasets used and analyzed during the current study are available from the corresponding author upon reasonable request.

## Abbreviations

aHRs: Adjusted hazard ratios
AIP: Atherogenic index of plasma
BMI: Body mass index
CIs: Confidence intervals
CumAIP: Cumulative atherogenic index of plasma
CumCRP: Cumulative high-sensitivity C-reactive protein
CVD: Cardiovascular disease
eGFR: Estimated glomerular filtration rate
FBG: Fasting blood glucose
HDL-C: High-density lipoprotein cholesterol
HRs: Hazard ratios
hsCRP: High-sensitivity C-reactive protein
IFG: Impaired fasting glucose
INTm: Multiplicative interaction
IQR: Interquartile range
IR: Insulin resistance
LDL-C: Low-density lipoprotein cholesterol
SBP: Systolic blood pressure
SD: Standard deviation
T2D: Type 2 diabetes
TC: Total cholesterol
TG: Triglycerides

## References

[1] Saeedi P, Petersohn I, Salpea P, Malanda B, Karuranga S, Unwin N, Colagiuri S, Guariguata L, Motala AA, Ogurtsova K (2019). Global and regional diabetes prevalence estimates for 2019 and projections for 2030 and 2045: results from the International Diabetes Federation Diabetes Atlas, 9(th) edition. Diabetes Res Clin Pract.

[2] Einarson T, Acs A, Ludwig C, Panton U (2018). Prevalence of cardiovascular disease in type 2 diabetes: a systematic literature review of scientific evidence from across the world in 2007-2017. Cardiovasc Diabetol.

[3] Khoo CM, Deerochanawong C, Chan SP, Matawaran B, Sheu WH, Chan J, Mithal A, Luk A, Suastika K, Yoon KH (2021). Use of sodium-glucose co-transporter-2 inhibitors in Asian patients with type 2 diabetes and kidney disease: an Asian perspective and expert recommendations. Diabetes Obes Metab.

[4] Tancredi M, Rosengren A, Svensson AM, Kosiborod M, Pivodic A, Gudbjörnsdottir S, Wedel H, Clements M, Dahlqvist S, Lind M (2015). Excess mortality among persons with type 2 diabetes. N Engl J Med.

[5] Griffin SJ, Borch-Johnsen K, Davies MJ, Khunti K, Rutten GE, Sandbæk A, Sharp SJ, Simmons RK, van den Donk M, Wareham NJ (2011). Effect of early intensive multifactorial therapy on 5-year cardiovascular outcomes in individuals with type 2 diabetes detected by screening (ADDITION-Europe): a cluster-randomised trial. Lancet.

[6] Chan J, Lim L, Wareham N, Shaw J, Orchard T, Zhang P, Lau E, Eliasson B, Kong A, Ezzati M (2021). The Lancet Commission on diabetes: using data to transform diabetes care and patient lives. Lancet (London, England).

[7] Jakubiak GK, Cieślar G, Stanek A (2022). Nitrotyrosine, nitrated lipoproteins, and cardiovascular dysfunction in patients with type 2 diabetes: what do we know and what remains to be explained?. Antioxidants (Basel).

[8] Taskinen MR (2003). Diabetic dyslipidaemia: from basic research to clinical practice. Diabetologia.

[9] Biondi-Zoccai GG, Abbate A, Liuzzo G, Biasucci LM (2003). Atherothrombosis, inflammation, and diabetes. J Am Coll Cardiol.

[10] Hansen SEJ, Madsen CM, Varbo A, Nordestgaard BG (2019). Low-grade inflammation in the association between mild-to-moderate hypertriglyceridemia and risk of acute pancreatitis: a study of more than 115000 individuals from the general population. Clin Chem.

[11] Eguchi K, Nagai R (2017). Islet inflammation in type 2 diabetes and physiology. J Clin Invest.

[12] Olefsky JM, Glass CK (2010). Macrophages, inflammation, and insulin resistance. Annu Rev Physiol.

[13] Bertoni A, Burke G, Owusu J, Carnethon M, Vaidya D, Barr R, Jenny N, Ouyang P, Rotter J (2010). Inflammation and the incidence of type 2 diabetes: the Multi-Ethnic Study of Atherosclerosis (MESA). Diabetes care.

[14] Glass CK, Olefsky JM (2012). Inflammation and lipid signaling in the etiology of insulin resistance. Cell Metab.

[15] Saltiel AR, Olefsky JM (2017). Inflammatory mechanisms linking obesity and metabolic disease. J Clin Invest.

[16] McGillicuddy FC, de la Llera MM, Hinkle CC, Joshi MR, Chiquoine EH, Billheimer JT, Rothblat GH, Reilly MP (2009). Inflammation impairs reverse cholesterol transport in vivo. Circulation.

[17] Chen S, Shimada K, Crother TR, Erbay E, Shah PK, Arditi M (2018). Chlamydia and lipids engage a common signaling pathway that promotes atherogenesis. J Am Coll Cardiol.

[18] Desikan R, Schork A, Wang Y, Thompson W, Dehghan A, Ridker P, Chasman D, McEvoy L, Holland D, Chen C (2015). Polygenic overlap between C-reactive protein, plasma lipids, and Alzheimer disease. Circulation.

[19] Zhao M, Song L, Sun L, Wang M, Wang C, Yao S, Li Y, Yun C, Zhang S, Sun Y (2021). Associations of type 2 diabetes onset age with cardiovascular disease and mortality: the Kailuan Study. Diabetes care.

[20] Zheng M, Zhang X, Chen S, Song Y, Zhao Q, Gao X, Wu S (2020). Arterial stiffness preceding diabetes: a longitudinal study. Circ Res.

[21] Fox CS, Golden SH, Anderson C, Bray GA, Burke LE, de Boer IH, Deedwania P, Eckel RH, Ershow AG, Fradkin J (2015). Update on prevention of cardiovascular disease in adults with type 2 diabetes mellitus in light of recent evidence: a scientific statement from the American Heart Association and the American Diabetes Association. Diabetes Care.

[22] DeGoma EM, French B, Dunbar RL, Allison MA, Mohler ER, Budoff MJ (2012). Intraindividual variability of C-reactive protein: the Multi-Ethnic Study of Atherosclerosis. Atherosclerosis.

[23] Ridker P (2016). A test in context: high-sensitivity C-reactive protein. J Am Coll Cardiol.

[24] Whelton PK, Carey RM, Aronow WS, Casey DE, Collins KJ, Dennison Himmelfarb C, DePalma SM, Gidding S, Jamerson KA, Jones DW (2018). 2017 ACC/AHA/AAPA/ABC/ACPM/AGS/APhA/ASH/ASPC/NMA/PCNA Guideline for the Prevention, Detection, Evaluation, and Management of High Blood Pressure in Adults: a report of the American College of Cardiology/American Heart Association Task Force on Clinical Practice Guidelines. J Am Coll Cardiol.

[25] Levey AS, Stevens LA, Schmid CH, Zhang YL, Castro AF, Feldman HI, Kusek JW, Eggers P, Van Lente F, Greene T (2009). A new equation to estimate glomerular filtration rate. Ann Intern Med.

[26] Izumida T, Nakamura Y, Hino Y, Ishikawa S (2020). Combined effect of small dense low-density lipoprotein cholesterol (sdLDL-C) and remnant-like particle cholesterol (RLP-C) on low-grade inflammation. J Atheroscler Thromb.

[27] Saeed A, Feofanova EV, Yu B, Sun W, Virani SS, Nambi V, Coresh J, Guild CS, Boerwinkle E, Ballantyne CM (2018). Remnant-like particle cholesterol, low-density lipoprotein triglycerides, and incident cardiovascular disease. J Am Coll Cardiol.

[28] Mooradian AD (2009). Dyslipidemia in type 2 diabetes mellitus. Nat Clin Pract Endocrinol Metab.

[29] Hsu H, Hsu P, Cheng MH, Ito Y, Kanda E, Schaefer EJ, Ai M (2019). Lipoprotein subfractions and glucose homeostasis in prediabetes and diabetes in Taiwan. J Atheroscler Thromb.

[30] Yan Y, Li S, Liu Y, Bazzano L, He J, Mi J, Chen W (2019). Temporal relationship between inflammation and insulin resistance and their joint effect on hyperglycemia: the Bogalusa Heart Study. Cardiovasc Diabetol.

[31] Hage FG, Szalai AJ (2007). C-reactive protein gene polymorphisms, C-reactive protein blood levels, and cardiovascular disease risk. J Am Coll Cardiol.

[32] Verma S, Mathew V, Farkouh M (2018). Targeting inflammation in the prevention and treatment of type 2 diabetes: insights from CANTOS. J Am Coll Cardiol.

[33] Duncan B, Schmidt M, Pankow J, Ballantyne C, Couper D, Vigo A, Hoogeveen R, Folsom A, Heiss G (2003). Low-grade systemic inflammation and the development of type 2 diabetes: the atherosclerosis risk in communities study. Diabetes.

[34] Johnson AR, Milner JJ, Makowski L (2012). The inflammation highway: metabolism accelerates inflammatory traffic in obesity. Immunol Rev.

[35] Šestan M, Marinović S, Kavazović I, Cekinović Đ, Wueest S, Turk Wensveen T, Brizić I, Jonjić S, Konrad D, Wensveen FM (2018). Virus-induced interferon-γ causes insulin resistance in skeletal muscle and derails glycemic control in obesity. Immunity.

[36] Chen S, de Craen A, Raz Y, Derhovanessian E, Vossen A, Westendorp R, Pawelec G, Maier A (2012). Cytomegalovirus seropositivity is associated with glucose regulation in the oldest old. Results from the Leiden 85-plus Study. Immun Ageing.

[37] Kraus W, Bhapkar M, Huffman K, Pieper C, Krupa Das S, Redman L, Villareal D, Rochon J, Roberts S, Ravussin E (2019). 2 years of calorie restriction and cardiometabolic risk (CALERIE): exploratory outcomes of a multicentre, phase 2, randomised controlled trial. Lancet Diabetes Endocrinol.

[38] Shivakoti R, Biggs ML, Djoussé L, Durda PJ, Kizer JR, Psaty B, Reiner AP, Tracy RP, Siscovick D, Mukamal KJ (2022). Intake and sources of dietary fiber, inflammation, and cardiovascular disease in older US adults. JAMA Netw Open.

[39] Healy G, Winkler E, Owen N, Anuradha S, Dunstan D (2015). Replacing sitting time with standing or stepping: associations with cardio-metabolic risk biomarkers. Eur Heart J.

[40] Messner B, Bernhard D (2014). Smoking and cardiovascular disease: mechanisms of endothelial dysfunction and early atherogenesis. Arterioscler Thromb Vasc Biol.

