# Supplementary material for: Temporal relationship between atherogenic dyslipidemia and inflammation and their joint cumulative effect on type 2 diabetes onset: a longitudinal cohort study
Source: BMC Med. 2023 Jan 24;21:31. doi: 10.1186/s12916-023-02729-6 (PMC9870774; doi:10.1186/s12916-023-02729-6)
Supplement: Supplementary file 1 — Additional file 1: Table S1. Number of participants and participations in the follow-up visits. Table S2. Value of CumAIP, mean AIP, CumCRP, and mean hsCRP during the exposure period. Table S3. Multivariable adjusted cross-lagged standard regression coefficient of hsCRP and AIP (n=52,225). Table S4. Pearson correlation coefficients between log-transformed hsCRP and HOMA-IR at baseline and follow-up in the total cohort and subgroups, adjusted for covariates. Table S5. CumCRP-associated risk for incident type 2 diabetes. Table S6. Type 2 diabetes risk with co-exposure stratified by CumCRP thresholds (1, 3 mg/L) and CumAIP (median) relative to different references. Table S7. Sensitivity analyses of incidence of type 2 diabetes with co-exposure stratified by CumCRP thresholds (1, 3 mg/L) and CumAIP (median). Table S8. Sensitivity analysis of CumAIP-associated type 2 diabetes risks in the entire cohort and across the cumulative CRP thresholds (1, 3 mg/L) using the ICPHREG procedure. Table S9. Sensitivity analysis of CumCRP-associated T2D risks in the entire cohort using the ICPHREG procedure. Table S10. Sensitivity analysis of co-exposure–associated type 2 diabetes risks in the entire cohort using the ICPHREG procedure. Table S11. CumAIP–associated type 2 diabetes risks in the entire cohort and across the cumulative CRP thresholds (1, 3 mg/L) with adjustment for time-varying covariates. Table S12. CumCRP-associated type 2 diabetes risks with adjustment for time-varying covariates. Table S13. The co-exposure-associated type 2 diabetes risks with adjustment for time-varying covariates. Table S14. Association between co-exposure to CumAIP and CumCRP and type 2 diabetes incidence stratified by sex. Table S15. Association between co-exposure to CumAIP and CumCRP and type 2 diabetes incidence stratified by IFG status in exposure period. Table S16. Association between co-exposure to CumAIP and CumCRP and type 2 diabetes incidence stratified by baseline blood pressure. Table S17 [file 12916_2023_2729_MOESM1_ESM.docx]

Additional File 1

Contents

[Table S1. Number of participants and participations in the follow-up visits 2](#_Toc121838860)

[Table S2. Value of CumAIP, mean AIP, CumCRP, and mean hsCRP during the exposure period 2](#_Toc121838861)

[Table S3. Multivariable adjusted cross-lagged standard regression coefficient of hsCRP and AIP (n=52,225) 3](#_Toc121838862)

[Table S4. Pearson correlation coefficients between log-transformed hsCRP and HOMA-IR at baseline and follow-up in the total cohort and subgroups, adjusted for covariates 3](#_Toc121838863)

[Table S5. CumCRP-associated risk for incident type 2 diabetes 4](#_Toc121838864)

[Table S6. Type 2 diabetes risk with co-exposure stratified by CumCRP thresholds (1, 3 mg/L) and CumAIP (median) relative to different references 5](#_Toc121838865)

[Table S7. Sensitivity analyses of incidence of type 2 diabetes with co-exposure stratified by CumCRP thresholds (1, 3 mg/L) and CumAIP (median) 6](#_Toc121838866)

[Table S8. Sensitivity analysis of CumAIP-associated type 2 diabetes risks in the entire cohort and across the cumulative CRP thresholds (1, 3 mg/L) using the ICPHREG procedure 8](#_Toc121838867)

[Table S9. Sensitivity analysis of CumCRP-associated T2D risks in the entire cohort using the ICPHREG procedure 10](#_Toc121838868)

[Table S10. Sensitivity analysis of co-exposure‒associated type 2 diabetes risks in the entire cohort using the ICPHREG procedure 11](#_Toc121838869)

[Table S11. CumAIP‒associated type 2 diabetes risks in the entire cohort and across the cumulative CRP thresholds (1, 3 mg/L) with adjustment for time-varying covariates 12](#_Toc121838870)

[Table S12 CumCRP-associated type 2 diabetes risks with adjustment for time-varying covariates 14](#_Toc121838871)

[Table S13 The co-exposure-associated type 2 diabetes risks with adjustment for time-varying covariates 15](#_Toc121838872)

[Table S14. Association between co-exposure to CumAIP and CumCRP and type 2 diabetes incidence stratified by sex 16](#_Toc121838873)

[Table S15. Association between co-exposure to CumAIP and CumCRP and type 2 diabetes incidence stratified by IFG status in exposure period 17](#_Toc121838874)

[Table S16. Association between co-exposure to CumAIP and CumCRP and type 2 diabetes incidence stratified by baseline blood pressure 18](#_Toc121838875)

[Table S17. Association between co-exposure to CumAIP and CumCRP and type 2 diabetes incidence stratified by dyslipidemia status 20](#_Toc121838876)

[Table S18. BasAIP-associated T2D in the entire cohort and across the baseline hsCRP thresholds (1,3 mg/L) 22](#_Toc121838877)

[Table S19. Type 2 diabetes risk on co-exposure stratified by BasCRP thresholds (1, 3 mg/L) and BasAIP (median) 24](#_Toc121838878)

[Fig. S1. Cross-lagged analysis design panel 25](#_Toc121838879)

# Table S1. Number of participants and participations in the follow-up visits

| **No. of participation in the follow-up visits** | **No. of participants** | **Health examinations** | **No. of participants** |
| --- | --- | --- | --- |
| 1 | 5380 | Visit_2012/2013 | 38710 |
| 2 | 9003 | Visit_2014/2015 | 31418 |
| 3 | 13300 | Visit_2016/2017 | 30074 |
| 4 | 15677 | Visit_2018/2020 | 25789 |

# Table S2. Value of CumAIP, mean AIP, CumCRP, and mean hsCRP during the exposure period

|  | **Mean (SD)** | **MAX** | **MIN** | **P25** | **P50** | **P75** |
| --- | --- | --- | --- | --- | --- | --- |
| CumAIP ^a^ | -0.1379 (0.26580) | 3.908 | -2.2936 | -0.5496 | -0.0701 | 0.2277 |
| mAIP ^b^ | -0.1332 (0.5783) | 3.4616 | -2.0184 | -0.5322 | -0.1633 | 0.2260 |
| CumCRP ^c^ | 2.72 (4.02) | 132.47 | 0.009 | 0.82 | 1.53 | 2.99 |
| mCRP ^d^ | 2.48 (3.45) | 89.27 | 0.01 | 0.76 | 1.40 | 2.77 |

^a^ CumAIP: cumulative atherogenic index of plasma in the exposure period.

^b^ mAIP: mean value of AIP in the three transient measures in the exposure period.

^c^ CumCRP: cumulative high-sensitivity C-reactive protein in the exposure period.

^d^ mCRP: mean value of hs-CRP in the three transient measures in the exposure period.

# Table S3. Multivariable adjusted cross-lagged standard regression coefficient of hsCRP and AIP (n=52,225)

|  | **R^2^ of hsCRP** | **R^2^ of AIP** | **AIP_2006/2007 to AIP_2010/2011** | **HsCRP_2006/2007 to HsCRP_2010/2011** | **AIP_2006/2007 to HsCRP_2010/2011** | **HsCRP_2006/2007 to AIP_2010/2011** | ***P* value** |
| --- | --- | --- | --- | --- | --- | --- | --- |
| Crude model | 0.0547 | 0.3874 | 0.26585 (0.6067,0.6182) | 0.2350 (0.2262,0.2439) | -0.0138 (-0.0230 to -0.0046) | 0.0632 (0.0554,0.0709) | P<0.001 |
| Model 1 | 0.0357 | 0.3313 | 0.5666 (0.5604,0.5728) | 0.1868 (0.1778,0.1957) | -0.0380 (-0.0473 to -0.0288) | 0.0781 (0.0702,0.0861) | P<0.001 |
| Model 2 | 0.0327 | 0.3274 | 0.5640 (0.5578,0.5702) | 0.1797 (0.1707,0.1887) | -0.0293 (-0.0385 to -0.0201) | 0.0740 (0.0659,0.0820) | P<0.001 |

Model 1: adjusted for age (continuous), sex, education, smoking status, drinking status, physical activities, BMI (continuous);

Model 2: further adjusted for FBG (continuous), SBP (continuous), TC (continuous), eGFR (categorical), antihypertensives (yes or no), and lipid-lowering drugs (yes or no) measured in 2006/2007 and the time intervals between examinations.

Abbreviations: AIP, atherogenic index of plasma; BMI, body mass index; eGFR, estimated glomerular filtration rate; FBG, fasting blood glucose; HsCRP, high-sensitivity C-reactive protein; SBP, systolic blood pressure; TC, total cholesterol.

# Table S4. Pearson correlation coefficients between log-transformed hsCRP and HOMA-IR at baseline and follow-up in the total cohort and subgroups, adjusted for covariates

| **Variables** | **HsCRP_2006/2007** | **AIP_2006/2007** | **HsCRP_2010/2011** |
| --- | --- | --- | --- |
| AIP_2006/2007 | 0.0408 (0.0324, 0.0492)* | - | - |
| HsCRP_2010/2011 | 0.1605 (0.1523, 0.1687)* | -0.0191 (-0.0275, -0.0107)* | - |
| AIP_2010/2011 | 0.0895 (0.0812, 0.0979)* | 0.5106 (0.5044, 0.5168)* | 0.0314 (0.0230, 0.0398)^*^ |

Covariates included in the models were age (continuous), sex, education, smoking, drinking status, physical activities, BMI (continuous), FBG (continuous), SBP (continuous), TC (continuous), eGFR (categorical), antihypertensives (yes or no), lipid-lowering drugs (yes or no) measured in 2006/2007, and the time intervals between 2006/2007 and 2010/2011.

“^*^” indicates a two-tailed P < 0.001.

# Table S5. CumCRP-associated risk for incident type 2 diabetes

|  | **CumCRP, AHRs (95% CIs)** | | | **logCumCRP per SD**^*^ | ***P* for trend** |
| --- | --- | --- | --- | --- | --- |
|  | **CumCRP<1 mg/L** | **1≤CumCRP<3 mg/L** | **CumCRP≥3 mg/L** |  |  |
| Event/Total | 1138/14011 | 2401/18551 | 1353/10798 |  |  |
| Incidence rate | 11.34 | 18.49 | 21.35 |  |  |
| Unadjusted model | Reference | 1.64 (1.53,1.76) | 1.89 (1.76,2.05) | 1.26 (1.23,1.30) | <0.001 |
| Model 1 | Reference | 1.38 (1.28,1.48) | 1.42 (1.30,1.54) | 1.13 (1.10,1.17) | <0.001 |
| Model 2 | Reference | 1.34 (1.24,1.44) | 1.41 (1.30,1.53) | 1.16 (1.13,1.19) | <0.001 |
| Model 3 | Reference | 1.26 (1.17,1.35) | 1.32 (1.20,1.45) | 1.12 (1.09,1.16) | <0.001 |

Model 1: adjusted for sex, education, smoking status, drinking status, physical activities, family history of diabetes, BMI (continuous);

Model 2: model 1+FBG (continuous), TC (continuous), logTG (continuous), blood pressure (categorical), eGFR (categorical), antihypertensives (yes or no), lipid-lowering drugs (yes or no);

Model 3: model 2 + fatty liver (categorical).

The incidence rate is per 1,000 person-years.

^*^ logCumCRP per SD, hazard ratio for per SD (0.4279) change in logCumCRP.

Abbreviations: BMI, body mass index; CumCRP, cumulative high-sensitivity C-reactive protein; eGFR, estimated glomerular filtration rate; FBG, fasting blood glucose; AHRs, average hazard ratios; SD, standard deviation; TC, total cholesterol; TG, triglyceride.

# Table S6. Type 2 diabetes risk with co-exposure stratified by CumCRP thresholds (1, 3 mg/L) and CumAIP (median) relative to different references

|  | **Combination of CumCRP and CumAIP, HRs (95% CIs)** | | | | | |
| --- | --- | --- | --- | --- | --- | --- |
|  | **CumAIP<-0.0701 & 1≤CumCRP<3 mg/L** | **CumAIP<-0.0701 & CumCRP<1 mg/L** | **CumAIP<-0.0701 & CumCRP≥3 mg/L** | **CumAIP≥-0.0701 & CumCRP<1 mg/L** | **CumAIP≥-0.0701 & 1≤CumCRP<3 mg/L** | **CumAIP≥-0.0701 & CumCRP≥3 mg/L** |
| Model 1 | Reference | 0.62 (0.55,0.71) | 0.97 (0.93,1.08) | 1.13 (1.02,1.23) | 1.35 (1.26,1.57) | 1.45 (1.38,1.64) |
| Model 2 | Reference | 0.67 (0.60,0.76) | 1.02 (0.91,1.14) | 1.18 (1.06,1.30) | 1.42 (1.30,1.55) | 1.59 (1.45,1.75) |
| Model 3 | Reference | 0.69 (0.62,0.78) | 1.00 (0.90,1.12) | 1.14 (1.03,1.26) | 1.30 (1.19,1.42) | 1.42 (1.29,1.56) |
|  | **CumAIP≥-0.0701 & CumCRP<1 mg/L** | **CumAIP<-0.0701 & CumCRP<1 mg/L** | **CumAIP<-0.0701 & 1≤CumCRP<3 mg/L** | **CumAIP<-0.0701 & CumCRP≥3 mg/L** | **CumAIP≥-0.0701 & 1≤CumCRP<3 mg/L** | **CumAIP≥-0.0701 & CumCRP≥3 mg/L** |
| Model 1 | Reference | 0.51 (0.45,0.57) | 0.78 (0.71,0.87) | 0.75 (0.67,0.84) | 1.22 (1.12,1.34) | 1.29 (1.17,1.43) |
| Model 2 | Reference | 0.57 (0.51,0.64) | 0.85 (0.77,0.94) | 0.87 (0.77,0.98) | 1.21 (1.11,1.32) | 1.36 (1.23,1.49) |
| Model 3 | Reference | 0.61 (0.54,0.69) | 0.88 (0.79,0.97) | 0.88 (0.78,0.99) | 1.14 (1.04,1.25) | 1.24 (1.13,1.37) |
|  | **CumAIP≥-0.0701 & 1≤CumCRP<3 mg/L** | **CumAIP<-0.0701 & CumCRP<1 mg/L** | **CumAIP<-0.0701 & 1≤CumCRP<3 mg/L** | **CumAIP<-0.0701 & CumCRP≥3 mg/L** | **CumAIP≥-0.0701 & CumCRP<1 mg/L** | **CumAIP≥-0.0701 & CumCRP≥3 mg/L** |
| Model 1 | Reference | 0.42 (0.37,0.46) | 0.64 (0.59,0.70) | 0.61 (0.55,0.68) | 0.82 (0.75,0.90) | 1.06 (0.98,1.14) |
| Model 2 | Reference | 0.47 (0.42,0.53) | 0.70 (0.65,0.77) | 0.72 (0.65,0.80) | 0.83 (0.76,0.90) | 1.12 (1.04,1.21) |
| Model 3 | Reference | 0.53 (0.48,0.60) | 0.77 (0.71,0.84) | 0.77 (0.70,0.86) | 0.88 (0.80,0.96) | 1.09 (1.01,1.18) |
|  | **CumAIP≥-0.0701 & CumCRP≥3 mg/L** | **CumAIP<-0.0701 & CumCRP<1 mg/L** | **CumAIP<-0.0701 & 1≤CumCRP<3 mg/L** | **CumAIP<-0.0701 & CumCRP≥3 mg/L** | **CumAIP≥-0.0701 & CumCRP<1 mg/L** | **CumAIP≥-0.0701 & 1≤CumCRP<3 mg/L** |
| Model 1 | Reference | 0.39 (0.35,0.44) | 0.60 (0.55,0.66) | 0.58 (0.52,0.64) | 0.77 (0.70,0.85) | 0.94 (0.87,1.02) |
| Model 2 | Reference | 0.42 (0.38,0.47) | 0.63 (0.57,0.69) | 0.64 (0.57,0.71) | 0.74 (0.67,0.81) | 0.89 (0.83,0.96) |
| Model 3 | Reference | 0.49 (0.44,0.55) | 0.71 (0.64,0.78) | 0.71 (0.63,0.79) | 0.80 (0.73,0.89) | 0.92 (0.85,0.99) |

Model 1: adjusted for age (continuous), sex, education, smoking status, drinking status, physical activities, family history of diabetes, BMI (continuous);

Model 2: model 1 + FBG (continuous), TC (continuous), blood pressure (categorical), eGFR (categorical), antihypertensives (yes or no), lipid-lowering drugs (yes or no);

Model 3: model 2+ fatty liver (categorical).

Abbreviations: CumAIP, cumulative atherogenic index of plasma; CumCRP, cumulative high-sensitivity C-reactive protein; HR, hazard ratio; BMI, body mass index; FBG, fasting blood glucose; TC, total cholesterol; eGFR, estimated glomerular filtration rate.

# Table S7. Sensitivity analyses of incidence of type 2 diabetes with co-exposure stratified by CumCRP thresholds (1, 3 mg/L) and CumAIP (median)

|  | **Combination of CumCRP and CumAIP, HRs (95% CIs)** | | | | | |
| --- | --- | --- | --- | --- | --- | --- |
|  | **CumAIP<-0.0701** & **CumCRP<1 mg/L** | **CumAIP<-0.0701** & **1≤CumCRP<3 mg/L** | **CumAIP<-0.0701** & **CumCRP≥3 mg/L** | **CumAIP≥-0.0701** & **CumCRP<1 mg/L** | **CumAIP≥-0.0701** & **1≤CumCRP<3 mg/L** | **CumAIP≥-0.0701** & **CumCRP≥3 mg/L** |
| **Excluded baseline CVD (4744/40980)** | | | | | | |
| Model 1 | Reference | 1.56 (1.38,1.77) | 1.50 (1.31,1.72) | 2.03 (1.79,2.30) | 2.50 (2.24,2.81) | 2.71 (2.40,3.05) |
| Model 2 | Reference | 1.52 (1.34,1.71) | 1.55 (1.35,1.78) | 1.80 (1.59,2.04) | 2.20 (1.96,2.46) | 2.53 (2.24,2.86) |
| Model 3 | Reference | 1.63 (1.29,1.66) | 1.47 (1.29,1.69) | 1.69 (1.49,1.92) | 1.94 (1.73,2.18) | 2.18 (1.92,2.46) |
| *P* for INTm: CumAIP median × CumCRP thresholds (1, 3 mg/L) = 0.0134 | | | | | | |
| **Exclude events in the first follow-up visit (3623/41865)** | | | | | | |
| Model 1 | Reference | 1.62 (1.40,1.86) | 1.32 (1.12,1.55) | 2.31 (2.00,2.67) | 2.64 (2.32,3.01) | 2.52 (2.32,3.01) |
| Model 2 | Reference | 1.58 (1.37,1.82) | 1.36 (1.16,1.61) | 2.10 (1.82,2.43) | 2.39 (2.09,2.72) | 2.41 (2.09,2.78) |
| Model 3 | Reference | 1.54 (1.33,1.77) | 1.31 (1.11,1.54) | 1.99 (1.74,2.30) | 2.14 (1.87,2.44) | 2.10 (1.82,2.43) |
| *P* for INTm: CumAIP median × CumCRP thresholds (1, 3 mg/L) = 0.0026 | | | | | | |
| **Exclude any hsCRP level≥10 mg/L in the exposure visits (2531/39124)** | | | | | | |
| Model 1 | Reference | 1.45 (1.30,1.62) | 1.43 (1.24,1.65) | 1.90 (1.69,2.14) | 2.26 (2.13,2.62) | 2.50 (2.21,2.81) |
| Model 2 | Reference | 1.41 (1.26,1.57) | 1.44 (1.25,1.66) | 1.69 (1.50,1.90) | 2.07 (2.83,2.30) | 2.32 (2.05,2.62) |
| Model 3 | Reference | 1.36 (1.22,1.52) | 1.37 (1.19,1.58) | 1.57 (1.40,1.77) | 1.81 (1.63,2.02) | 1.98 (1.75,2.24) |
| *P* for INTm: CumAIP median × CumCRP thresholds (1, 3 mg/L) = 0.0517 | | | | | | |
| **Excluded missing data (5099/43157)** | | | | | | |
| Model 1 | Reference | 1.53 (1.31,1.74) | 1.54 (1.31,1.69) | 1.98 (1.53,2.49) | 2.43 (2.17,2.72) | 2.53 (2.25,2.88) |
| Model 2 | Reference | 1.49 (1.32,1.68) | 1.52 (1.33,1.74) | 1.75 (1.55,1.98) | 2.12 (1.90,2.36) | 2.37 (2.11,2.66) |
| Model 3 | Reference | 1.44 (1.28,1.62) | 1.45 (1.27,1.65) | 1.64 (1.45,1.84) | 1.87 (1.68,2.09) | 2.04 (1.81,2.29) |
| *P* for INTm: CumAIP median × CumCRP thresholds (1, 3 mg/L) = 0.0401 | | | | | | |

Model 1: adjusted for age (continuous), sex, education, smoking status, drinking status, physical activities, family history of diabetes, BMI (continuous);

Model 2: model 1 + FBG (continuous), TC (continuous), blood pressure (categorical), eGFR (categorical), antihypertensives (yes or no), lipid-lowering drugs (yes or no);

Model 3: model 2+ fatty liver (categorical).

Abbreviations: BMI, body mass index; CIs, confidence intervals; CumAIP, cumulative atherogenic index of plasma; CumCRP, cumulative high-sensitivity C-reactive protein; CVD, cardiovascular diseases; eGFR, estimated glomerular filtration rate; FBG, fasting blood glucose; HRs, hazard ratios; INTm, multiplicative interaction; SD, standard deviation; TC, total cholesterol.

# Table S8. Sensitivity analysis of CumAIP-associated type 2 diabetes risks in the entire cohort and across the cumulative CRP thresholds (1, 3 mg/L) using the ICPHREG procedure

|  | **CumAIP, HRs (95% CIs)** | | | | **Per SD** | ***P* for trend** |
| --- | --- | --- | --- | --- | --- | --- |
|  | **Quartile1** | **Quartile 2** | **Quartile 3** | **Quartile 4** |  |  |
| Entire cohort | | | | | | |
| Unadjusted model | Reference | 1.56 (1.41,1.72) | 2.18 (1.98,2.39) | 3.18 (2.91,3.47) | 1.47 (1.43,1.50) | <0.0001 |
| Model 1 | Reference | 1.38 (1.25,1.52) | 1.78 (1.62,1.96) | 2.42 (2.21,2.65) | 1.36 (1.32,1.39) | <0.0001 |
| Model 2 | Reference | 1.26 (1.14,1.39) | 1.56 (1.42,1.71) | 2.03 (1.85,2.23) | 1.28 (1.24,1.31) | <0.0001 |
| Model 3 | Reference | 1.23 (1.11,1.36) | 1.44 (1.31,1.59) | 1.76 (1.60,1.93) | 1.20 (1.17,1.24) | <0.0001 |
| CumCRP<1 mg/L | | | | | | |
| Unadjusted model | Reference | 1.59 (1.31,1.93) | 2.43 (2.02,2.91) | 3.71 (3.12, 4.42) | 1.61 (1.52,1.69) | <0.0001 |
| Model 1 | Reference | 1.38 (1.14,1.68) | 1.94 (1.61,2.34) | 2.82 (2.35,3.40) | 1.48 (1.40,1.57) | <0.0001 |
| Model 2 | Reference | 1.26 (1.04,1.53) | 1.59 (1.31,1.92) | 2.31 (1.91,2.78) | 1.38 (1,30,1.46) | <0.0001 |
| Model 3 | Reference | 1.23 (1.01,1.50) | 1.49 (1.23,1.80) | 2.01 (1.66,2.43) | 1.29 (1.21,1.37) | <0.0001 |
| 1≤CumCRP<3 mg/L | | | | | | |
| Unadjusted model | Reference | 1.41 (1.22, 1.63) | 1.84 (1.60, 2.11) | 2.63 (2.31,3.00) | 1.39 (1.34,1.44） | <0.0001 |
| Model 1 | Reference | 1.27 (1.10,1.47) | 1.56 (1.36,1.79) | 2.13 (1.86,2.44) | 1.31 (1.26,1.36) | <0.001 |
| Model 2 | Reference | 1.15 (1.00,1.33) | 1.37 (1.19,1.58) | 1.80 (1.57,2.06) | 1.24 (1.19,1.30) | <0.001 |
| Model 3 | Reference | 1.14 (0.99,1.32) | 1.31 (1.14,1.50) | 1.61 (1.41,1.85) | 1.18 (1.13,1.24) | <0.001 |
| CumCRP≥3 mg/L | | | | | | |
| Unadjusted model | Reference | 1.57 (1.31,1.88) | 2.19 (1.85,2.61) | 2.92 (2.52, 3.49) | 1.40 (1.34,1.46) | <0.0001 |
| Model 1 | Reference | 1.43 (1.19,1.72) | 1.86 (1.56,2.22) | 2.34 (1.98,2.76) | 1.30 (1.24,1.36) | <0.0001 |
| Model 2 | Reference | 1.35 (1.13,1.63) | 1.70 (1.43,2.03) | 2.01 (1.70,2.39) | 1.23 (1.17,1.29) | <0.0001 |
| Model 3 | Reference | 1.31 (1.09,1.57) | 1.59 (1.33,1.90) | 1.74 (1.47,2.07) | 1.16 (1.10,1.22) | <0.0001 |

Quartile 1: CumAIP<-0.2387; Quartile 2: -0.2387≤CumAIP< -0.0701; Quartile 3: -0.0701≤ CumAIP<0.0989; Quartile 4: CumAIP>0.0989.

Model 1: adjusted for baseline age, sex, education, smoking, drinking status, physical activities, family history of diabetes, BMI; Model 2: model 1+ FBG, TC, blood pressure (categorical), eGFR (categorical), antihypertensives, lipid-lowering drugs, logBasCRP (continuous, only in entire cohort); Model 3: model 2+fatty liver (categorical).

*Per SD, hazard ratio for per SD (0.2658) change in CumAIP.

Abbreviations: CumAIP, cumulative atherogenic index of plasma; CumCRP, cumulative high-sensitivity C-reactive protein; BMI, body mass index; FBG, fasting blood glucose; TC, total cholesterol; eGFR, estimated glomerular filtration rate; BasCRP: baseline high-sensitivity C-reactive protein.

# Table S9. Sensitivity analysis of CumCRP-associated T2D risks in the entire cohort using the ICPHREG procedure

|  | **CumCRP, AHRs (95% CIs)** | | | **logCumCRP per SD**^*^ | ***P* for trend** |
| --- | --- | --- | --- | --- | --- |
|  | **CumCRP<1 mg/L** | **1≤CumCRP<3 mg/L** | **CumCRP≥3 mg/L** |  |  |
| Unadjusted model | Reference | 1.63 (1.52,1.75) | 1.88 (1.74,2.03) | 1.26 (1.23,1.29) | <0.0001 |
| Model 1 | Reference | 1.38 (1.28,1.48) | 1.41 (1.31,1.53) | 1.13 (1.10,1.17) | <0.0001 |
| Model 2 | Reference | 1.30 (1.21,1.40) | 1.40 (1.29,1.52) | 1.14 (1.11,1.18) | <0.0001 |
| Model 3 | Reference | 1.24 (1.15,1.33) | 1.31 (1.21,1.43) | 1.12 (1.08,1.15) | <0.0001 |

Model 1: adjusted for sex, education, smoking status, drinking status, physical activities, family history of diabetes, BMI (continuous);

Model 2: model 1+FBG (continuous), TC (continuous), logTG (continuous), blood pressure (categorical), eGFR (categorical), antihypertensives (yes or no), lipid-lowering drugs (yes or no);

Model 3: model 2 + fatty liver (categorical).

* logCumCRP per SD, hazard ratio for per SD (0.4279) change in logCumCRP.

Abbreviations: CumAIP, cumulative atherogenic index of plasma; CumCRP, cumulative high-sensitivity C-reactive protein; BMI, body mass index; FBG, fasting blood glucose; TC, total cholesterol; TG, triglyceride; eGFR, estimated glomerular filtration rate.

# Table S10. Sensitivity analysis of co-exposure‒associated type 2 diabetes risks in the entire cohort using the ICPHREG procedure

|  | **Combination of CumCRP and CumAIP, HRs (95% CIs)** | | | | | |
| --- | --- | --- | --- | --- | --- | --- |
|  | **G1** | **G2** | **G3** | **G4** | **G5** | **G6** |
| Model(unadjusted) | Reference | 1.77 (1.57,1.99) | 1.90 (1.67,2.17) | 2.37 (2.10,2.68) | 3.25 (2.92,3.62) | 3.84 (3.43,4.29) |
| Model 1 | Reference | 1.54 (1.37,1.73) | 1.49 (1.30,1.70) | 1.99 (1.76,2.25) | 2.42 (2.17,2.71) | 2.55 (2.27,2.86) |
| Model 2 | Reference | 1.50 (1.33,1.69) | 1.53 (1.34,1.75) | 1.76 (1.56,1.99) | 2.13 (1.90,2.38) | 2.40 (2.14,2.70) |
| Model 3 | Reference | 1.45 (1.28,1.63) | 1.46 (1.28,1.67) | 1.66 (1.46,1.87) | 1.88 (1.68,2.10) | 2.10 (1.83,2.33) |

G1: CumAIP<-0.0701 & CumCRP<1 mg/L; G2: CumAIP<-0.0701 & 1≤CumCRP<3 mg/L; G3: CumAIP<-0.0701 & CumCRP≥3 mg/L; G4: CumAIP≥-0.0701 & CumCRP<1 mg/L; G5: CumAIP≥-0.0701 & 1≤CumCRP<3 mg/L; G6: CumAIP≥-0.0701 & CumCRP≥3 mg/L.

Model 1: adjusted for age, sex, education, smoking, drinking status, physical activities, family history of diabetes, and BMI;

Model 2: model 1 + FBG, TC, blood pressure (categorical), eGFR (categorical), antihypertensives, and lipid‒lowering drugs;

Model 3: model 2+ fatty liver (categorical).

Abbreviations: BMI, body mass index; CIs, confidence intervals; CumAIP, cumulative atherogenic index of plasma; CumCRP, cumulative high-sensitivity C-reactive protein; CVD, cardiovascular diseases; eGFR, estimated glomerular filtration rate; FBG, fasting blood glucose; HRs, hazard ratios; INTm, multiplicative interaction; SD, standard deviation; TC, total cholesterol.

# Table S11. CumAIP‒associated type 2 diabetes risks in the entire cohort and across the cumulative CRP thresholds (1, 3 mg/L) with adjustment for time-varying covariates

|  | **CumAIP, HRs (95% CIs)** | | | | **Per SD** | ***P* for trend** |
| --- | --- | --- | --- | --- | --- | --- |
|  | **Quartile1** | **Quartile 2** | **Quartile 3** | **Quartile 4** |  |  |
| Entire cohort | | | | | | |
| Model 1 | Reference | 1.43 (1.29,1.57) | 1.88 (1.71,2.06) | 2.58 (2.36,2.82) | 1.38 (1.34,1.41) | <0.0001 |
| Model 2 | Reference | 1.38 (1.25,1.53) | 1.84 (1.67,2.02) | 2.40 (1.20,2.63) | 1.34 (1.30,1.37) | <0.0001 |
| Model 3 | Reference | 1.34 (1.21,1.48) | 1.69 (1.54,1.86) | 2.05 (1.87,2.25) | 1.26 (1.23,1.30) | <0.0001 |
| Model 4 | Reference | 1.23 (1.11,1.35) | 1.48 (1.35,1.63) | 1.76 (1.60,1.93) | 1.20 (1.17,1.23) | <0.0001 |
| CumCRP<1 mg/L | | | | | | |
| Model 1 | Reference | 1.41 (1.16,1.71) | 1.98 (1.65,2.38) | 2.87 (2.39,3.449) | 1.48 (1.40,1.57) | <0.0001 |
| Model 2 | Reference | 1.35 (1.11,1.64) | 1.88 (1.56,2.27) | 2.71 (2.26,3.26) | 1.47 (1.39,1.56) | <0.0001 |
| Model 3 | Reference | 1.31 (1.08,1.59) | 1.73 (1.43,2.08) | 2.27 (1.88,2.74) | 1.37 (1.29,1.46) | <0.0001 |
| Model 4 | Reference | 1.21 (0.99,1.47) | 1.50 (1.24,1.81) | 1.93 (1.60,2.33) | 1.28 (1.20,1.36) | <0.0001 |
| 1≤CumCRP<3 mg/L | | | | | | |
| Model 1 | Reference | 1.33 (1.15,1.53) | 1.65 (1.44,1.90) | 2.27 (1.98,2.59) | 1.33 (1.28,1.38) | <0.001 |
| Model 2 | Reference | 1.31 (1.13,1.52) | 1.59 (1.39,1.83) | 2.14 (1.87,2.45) | 1.30 (1.25,1.35) | <0.001 |
| Model 3 | Reference | 1.27 (1.10,1.47) | 1.48 (1.29,1.71) | 1.86 (1.62,2.13) | 1.23 (1.18,1.28) | <0.001 |
| Model 4 | Reference | 1.15 (1.00,1.33) | 1.33 (1.15,1.52) | 1.60 (1.40,2.83) | 1.17 (1.12,1.22) | <0.001 |
| CumCRP≥3 mg/L | | | | | | |
| Model 1 | Reference | 1.44 (1.20,1.73) | 1.91 (1.60,2.27) | 2.38 (2.02,2.81) | 1.30 (1.24,1.36) | <0.0001 |
| Model 2 | Reference | 1.36 (1.13,1.63) | 1.80 (1.52,2.15) | 2.15 (1.82,2.54) | 1.25 (1.19,1.31) | <0.0001 |
| Model 3 | Reference | 1.32 (1.10,1.59) | 1.71 (1.43,2.04) | 1.94 (1.65,2.31) | 1.19 (1.14,1.25) | <0.0001 |
| Model 4 | Reference | 1.25 (1.04,1.51) | 1.52 (1.27,1.81) | 1.68 (1.42,1.99) | 1.14 (1.08,1.19) | <0.0001 |

*P*-INTm: AIP (quartiles)* CRP (1, 3 mg/L)=0.0102 when adjusting for time-varying covariates.

*P*-INTm: AIP (quartiles)* CRP(1, 3 mg/L)=0.0268 when adjusting for time-vaying covariates except for FBG (baseline).

Quartile 1: CumAIP<-0.2387; Quartile 2: -0.2387≤CumAIP<-0.0701; Quartile 3: -0.0701≤CumAIP<0.0989; Quartile 4: CumAIP≥0.0989.

Model 1: adjusted for age (time-varying), sex, education (time-varying), smoking (time-varying), drinking status (time-varying), physical activities (time-varying), family history of diabetes (time-varying), and BMI (time-varying);

Model 2: model 1 + FBG (time-varying), TC (time-varying), blood pressure (time-varying), eGFR (time-varying), antihypertensives (time-varying), and lipid‒lowering drugs (time-varying), hsCRP (time-varying, limited to the entire cohort)

Model 3: model 2+ fatty liver (time-varying)).

Model 4: Model 3+ baseline FBG instead of time-varying FBG.

Abbreviations: CumAIP, cumulative atherogenic index of plasma; CumCRP, cumulative high-sensitivity C-reactive protein; BMI, body mass index; FBG, fasting blood glucose; TC, total cholesterol; eGFR, estimated glomerular filtration rate; hsCRP: high-sensitivity C-reactive protein; INTm, multiplicative interaction.

# Table S12 CumCRP-associated type 2 diabetes risks with adjustment for time-varying covariates

|  | **CumCRP, AHRs (95% CIs)** | | | **logCumCRP per SD**^*^ | ***P* for trend** |
| --- | --- | --- | --- | --- | --- |
|  | **CumCRP<1 mg/L** | **1≤CumCRP<3 mg/L** | **CumCRP≥3 mg/L** |  |  |
| Unadjusted model | Reference | 1.64 (1.53,1.76) | 1.89 (1.76,2.05) | 1.26 (1.23,1.30) | <0.001 |
| Model 1 | Reference | 1.49 (1.38,1.60) | 1.66 (1.53,1.80) | 1.21(1.18,1.24) | <0.001 |
| Model 2 | Reference | 1.48 (1.37,1.59) | 1.62 (1.50,1.76) | 1.20 (1.16,1.23) | <0.001 |
| Model 3 | Reference | 1.38 (1.29,1.49) | 1.48 (1.36,1.60) | 1.16 (1.13,1.20) | <0.001 |
| Model 4 | Reference | 1.33 (1.23,1.44) | 1.53 (1.41,1.66) | 1.16 (1.13,1.20) | <0.001 |

Model 1: adjusted for age (time-varying), sex, education (time-varying), smoking (time-varying), drinking status (time-varying), physical activities (time-varying), family history of diabetes (time-varying), and BMI (time-varying);

Model 2: model 1 + FBG (time-varying), TC (time-varying), logTG (time-varying), blood pressure (time-varying), eGFR (time-varying), antihypertensives (time-varying), and lipid‒lowering drugs (time-varying).

Model 3: model 2+ fatty liver (time-varying)).

Model 4: Model 3+ baseline FBG instead of time-varying FBG.

Abbreviations: CumCRP, cumulative high-sensitivity C-reactive protein; AHR, average hazard ratio; BMI, body mass index; FBG, fasting blood glucose; TC, total cholesterol; TG, triglyceride; eGFR, estimated glomerular filtration rate.

# Table S13 The co-exposure-associated type 2 diabetes risks with adjustment for time-varying covariates

|  | **Combination of CumCRP and CumAIP, HRs (95% CIs)** | | | | | |
| --- | --- | --- | --- | --- | --- | --- |
|  | **G1** | **G2** | **G3** | **G4** | **G5** | **G6** |
| Model 1 | Reference | 1.65 (1.47,1.86) | 1.74 (1.52,1.99) | 2.08 (1.84,2.35) | 2.69 (2.41,3.00) | 3.09 (2.75,3.47) |
| Model 2 | Reference | 1.66 (1.48,1.87) | 1.75 (1.53,1.99) | 2.01 (1.78,2.28) | 2.60 (2.33,2.90) | 2.94 (2.62,3.30) |
| Model 3 | Reference | 1.58 (1.40,1.78) | 1.60 (1.40,1.83) | 1.85 (1.63,2.09) | 2.24 (2.00,2.50) | 2.44 (2.17,2.74) |
| Model 4 | Reference | 1.51 (1.34,1.70) | 1.65 (1.44,1.88) | 1.66 (1.47,1.88) | 1.97 (1.77,2.21) | 2.30 (2.05,2.59) |
| *P*-INTm: CumCRP (1, 3 mg/L) * CumAIP (median)= 0.0023 (Model 3);  *P*-INTm: CumCRP (1, 3 mg/L) * CumAIP (median)= 0.0079 (Model 4); | | | | | | |

G1: CumAIP<-0.0701 & CumCRP<1 mg/L; G2: CumAIP<-0.0701 & 1≤CumCRP<3 mg/L; G3: CumAIP<-0.0701 & CumCRP≥3 mg/L; G4: CumAIP≥-0.0701 & CumCRP<1 mg/L; G5: CumAIP≥-0.0701 & 1≤CumCRP<3 mg/L; G6: CumAIP≥-0.0701 & CumCRP≥3 mg/L.

Model 1: adjusted for age (time-varying), sex, education (time-varying), smoking (time-varying), drinking status (time-varying), physical activities (time-varying), family history of diabetes (time-varying), and BMI (time-varying);

Model 2: model 1 + FBG (time-varying), TC (time-varying), blood pressure (time-varying), eGFR (time-varying), antihypertensives (time-varying), and lipid‒lowering drugs (time-varying).

Model 3: model 2+ fatty liver (time-varying)).

Model 4: Model 3+ baseline FBG instead of time-varying FBG.

Abbreviations: BMI, body mass index; CIs, confidence intervals; CumAIP, cumulative atherogenic index of plasma; CumCRP, cumulative high-sensitivity C-reactive protein; CVD, cardiovascular diseases; eGFR, estimated glomerular filtration rate; FBG, fasting blood glucose; HRs, hazard ratios; INTm, multiplicative interaction; TC, total cholesterol.

# Table S14. Association between co-exposure to CumAIP and CumCRP and type 2 diabetes incidence stratified by sex

|  | | **Combination of CumCRP and CumAIP, HRs (95% CIs)** | | | | | | ***P* value for interaction** |
| --- | --- | --- | --- | --- | --- | --- | --- | --- |
|  | **CumCRP<1 mg/L**  **CumAIP<-0.0701** | | **1≤CumCRP<3 mg/L**  **CumAIP<-0.0701** | **CumCRP≥3 mg/L**  **CumAIP<-0.0701** | **CumCRP<1 mg/L**  **CumAIP≥-0.0701** | **1≤CumCRP<3 mg/L**  **CumAIP≥-0.0701** | **CumCRP≥3 mg/L**  **CumAIP≥-0.0701** |  |
| **Male** | | | | | | | | 0.0034 |
| Event/Total | 321/5353 | | 570/6173 | 363/3620 | 623/4969 | 1286/8113 | 812/4390 |  |
| Incidence rate | 8.36 | | 13.11 | 14.46 | 18.13 | 23.21 | 27.65 |  |
| Unadjusted model | Reference | | 1.57 (1.37,1.80) | 1.73 (1.49,2.01) | 2.15 (1.88,2.46) | 2.77 (2.45,3.13) | 3.31 (2.91,3.76) |  |
| Model 1 | Reference | | 1.41 (1.23,1.62) | 1.40 (1.20,1.63) | 1.90 (1.66,2.18) | 2.17 (1.91,2.46) | 2.33 (2.04,2.66) |  |
| Model 2 | Reference | | 1.38 (1.20,1.58) | 1.45 (1.24,1.68) | 1.72 (1.50,1.97) | 1.95 (1.72,2.21) | 2.24 (1.96,2.56) |  |
| Model 3 | Reference | | 1.34 (1.16,1.53) | 1.38 (1.19,1.61) | 1.63 (1.42,1.87) | 1.74 (1.54,1.98) | 1.95 (1.70,2.24) |  |
| **Female** | | | | | | | |  |
| Event/Total | 108/2733 | | 219/2412 | 126/1389 | 86/956 | 326/1853 | 278/1399 |  |
| Incidence rate | 5.23 | | 12.11 | 12.19 | 12.40 | 25.21 | 28.38 |  |
| Unadjusted model | Reference | | 2.34 (1.86,2.95) | 2.35 (1.81,3.03) | 2.36 (1.78,3.14) | 4.86 (3.91,6.04) | 5.48 (4.38,6.84) |  |
| Model 1 | Reference | | 1.94 (1.53,2.44) | 1.71 (1.31,2.22) | 1.83 (1.38,2.44) | 3.27 (2.61,4.09) | 3.25 (2.57,4.11) |  |
| Model 2 | Reference | | 1.83 (1.45,2.32) | 1.74 (1.34,2.27) | 1.60 (1.20,2.12) | 2.82 (2.22,3.57) | 2.82 (2.22,3.57) |  |
| Model 3 | Reference | | 1.75 (1.38,2.21) | 1.62 (1.24,2.11) | 1.46 (1.10,1.95) | 2.42 (1.92,3.05) | 2.38 (1.86,3.03) |  |

Model 1: adjusted for age (continuous), education, smoking, drinking status, physical exercise, family history of diabetes, BMI (continuous);

Model 2: Model 1+FBG (continuous), TC (continuous), blood pressure (categorical), eGFR (categorical), antihypertensives (yes or no), lipid-lowering drugs (yes or no);

Model 3: model 2+ fatty liver (categorical).

The incident rate is per 1,000 person-years.

Abbreviations: BMI, body mass index; CumAIP, cumulative atherogenic index of plasma; CumCRP, cumulative high-sensitivity C-reactive protein; eGFR, estimated glomerular filtration rate; FBG, fasting blood glucose; HR, hazard ratio; TC, total cholesterol.

# Table S15. Association between co-exposure to CumAIP and CumCRP and type 2 diabetes incidence stratified by IFG status in exposure period

|  | **Combination of CumCRP and CumAIP, HR (95% CI)** | | | | | | ***P* value for interaction** |
| --- | --- | --- | --- | --- | --- | --- | --- |
|  | **CumCRP<1 mg/L**  **CumAIP<-0.0701** | **1≤CumCRP<3 mg/L**  **CumAIP<-0.0701** | **CumCRP≥3 mg/L**  **CumAIP<-0.0701** | **CumCRP<1 mg/L**  **CumAIP≥-0.0701** | **1≤CumCRP<3 mg/L**  **CumAIP≥-0.0701** | **CumCRP≥3 mg/L**  **CumAIP≥-0.0701** |  |
| I**FG** | | | | | | | 0.0365 |
| Event/Total | 182/1030 | 327/1433 | 220/772 | 337/1168 | 765/2141 | 485/1153 |  |
| Incidence rate | 26.91 | 36.55 | 47.47 | 48.12 | 61.34 | 77.75 |  |
| Model 1 | Reference | 1.27 (1.06,1.52) | 1.45 (1.19,1.77) | 1.63 (1.38,1.98) | 1.91 (1.62,2.26) | 2.10 (1.76,2.50) |  |
| Model 2 | Reference | 1.26 (1.05,1.52) | 1.45 (1.19,1.77) | 1.61 (1.34,1.93) | 1.88 (1.60,2.22) | 2.04 (1.71,2.43) |  |
| Model 3 | Reference | 1.21 (1.01,1.45) | 1.36 (1.12,1.66) | 1.50 (1.25,1.80) | 1.65 (1.40,1.95) | 1.70 (1.42,2.04) |  |
| **Without IFG** | | | | | | |  |
| Event/Total | 247/7056 | 463/7152 | 269/4237 | 372/4757 | 847/7825 | 605/4636 |  |
| Incidence rate | 4.73 | 8.78 | 8.73 | 10.85 | 15.16 | 18.38 |  |
| Model 1 | Reference | 1.64 (1.40,1.92) | 1.48 (1.24,1.76) | 1.93 (1.64,2.27) | 2.42 (2.09,2.80) | 2.69 (2.30,3.13) |  |
| Model 2 | Reference | 1.62 (1.38,1.89) | 1.46 (1.23,1.74) | 1.86 (1.58,2.19) | 2.32 (2.01,2.69) | 2.58 (2.21,3.01) |  |
| Model 3 | Reference | 1.57 (1.34,1.83) | 1.41 (1.18,1.67) | 1.75 (1.48,2.06) | 2.05 (1.76,2.37) | 2.22 (1.90,2.60) |  |

Model 1: adjusted for age (continuous), sex, education, smoking status, drinking status, physical exercise, family history of diabetes, BMI (continuous);

Model 2: Model 1+TC (continuous), blood pressure (categorical), eGFR (categorical), antihypertensives (yes or no), lipid-lowering drugs (yes or no);

Model 3: model 2+ fatty liver (categorical).

Impaired fasting glucose (IFG) was defined as having an FBG concentration between 6.1-6.9 mmol/L while not using antidiabetic medication.

Abbreviations: IFG, impaired fasting glucose; CumAIP, cumulative atherogenic index of plasma; CumCRP, cumulative high-sensitivity C-reactive protein; HR, hazard ratio; BMI, body mass index; FBG, fasting blood glucose; TC, total cholesterol; eGFR, estimated glomerular filtration rate.

The incident rate is per 1,000 person-years.

# Table S16. Association between co-exposure to CumAIP and CumCRP and type 2 diabetes incidence stratified by baseline blood pressure

|  | | Combination of CumCRP and CumAIP, HR (95% CI) | | | | | | *P* value for interaction |
| --- | --- | --- | --- | --- | --- | --- | --- | --- |
|  | CumCRP<1 mg/L  CumAIP<-0.0701 | | 1≤CumCRP<3 mg/L  CumAIP<-0.0701 | CumCRP≥3 mg/L  CumAIP<-0.0701 | CumCRP<1 mg/L  CumAIP≥-0.0701 | 1≤CumCRP<3 mg/L  CumAIP≥-0.0701 | CumCRP≥3 mg/L  CumAIP≥-0.0701 |  |
| **Normal blood pressure ^a^** | | | | | | | | 0.0005 |
| Model 1 | Reference | | 1.73 (1.46,2.04) | 1.65 (1.36,1.99) | 2.30 (1.94,2.73) | 2.85 (2.44,3.33) | 3.08 (2.61,3.64) |  |
| Model 2 | Reference | | 1.66 (1.41,1.96) | 1.72 (1.43,2.08) | 2.04 (1.72,2.42) | 2.52 (2.15,2.94) | 2.90 (2.45,3.43) |  |
| Model 3 | Reference | | 1.61 (1.37,1.90) | 1.62 (1.34,1.95) | 1.92 (1.62,2.28) | 2.22 (1.89,2.59) | 2.46 (2.07,2.92) |  |
| **Grade I hypertension ^b^** | | | | | | | |  |
| Model 1 | Reference | | 1.32 (1.09,1.59) | 1.26 (1.02,1.54) | 1.56 (1.29,1.81) | 1.87 (1.58,2.22) | 1.94 (1.62,2.33) |  |
| Model 2 | Reference | | 1.30 (1.08,1.57) | 1.32 (1.07,1.62) | 1.43 (1.18,1.73) | 1.71 (1.44,2.03) | 1.84 (1.54,2.21) |  |
| Model 3 | Reference | | 1.26 (1.04,1.52) | 1.26 (1.03,1.55) | 1.33 (1.10,1.61) | 1.51 (1.27,1.80) | 1.60 (1.33,1.92) |  |
| **Grade II hypertension ^c^** | | | | | | | |  |
| Model 1 | Reference | | 1.08 (0.71,1.63) | 1.24 (0.81,1.91) | 1.50 (0.99,2.29) | 1.81 (1.25,2.62) | 2.00 (1.36,2.93) |  |
| Model 2 | Reference | | 1.17 (0.77,1.77) | 1.29 (0.84,1.98) | 1.45 (0.95,2.21) | 1.78 (1.23,2.59) | 2.11 (1.43,3.11) |  |
| Model 3 | Reference | | 1.14 (0.75,1.73) | 1.27 (0.83,1.97) | 1.39 (0.91,212) | 1.63 (1.20,2.38) | 1.87 (1.26,1.78) |  |
| **Grade III hypertension ^d^** | | | | | | | |  |
| Model 1 | Reference | | 1.55 (0.64, 3.74) | 0.34 (0.10,1.17) | 0.67 (0.21,2.16) | 0.76 (0.31,1.89) | 1.16 (0.46,2.93) |  |
| Model 2 | Reference | | 1.49 (0.61,3.64) | 0.47 (0.14,1.62) | 0.71 (0.22,2.33) | 0.88 (0.35,2.19) | 1.28 (0.49,3.33) |  |
| Model 3 | Reference | | 1.43 (0.57,3.57) | 0.49 (0.14,1.64) | 0.62 (0.19,2.06) | 0.78 (0.31,1.98) | 1.12 (0.43,2.95) |  |

Model 1: adjusted for age (continuous), sex, education, smoking status, drinking status, physical exercise, family history of diabetes, BMI (continuous);

Model 2: Model 1+ FBG (continuous), TC (continuous), eGFR (categorical), antihypertensives (yes or no), lipid-lowering drugs (yes or no);

Model 3: model 2+ fatty liver (categorical).

Abbreviations are the same as shown in Table S4.

^a^ Normal blood pressure: SBP<140 mmHg and DBP<90 mmHg;

^b^ Grade I hypertension: SBP≥140 but <160 mmHg or DBP≥90 mmHg but <100 mmHg;

^c^ Grade II hypertension: SBP≥160 mmHg but <180 mmHg or DBP≥100 mmHg but <110 mmHg;

^d^ Grade III hypertension: SBP≥180 mmHg or DBP≥110 mmHg.

# Table S17. Association between co-exposure to CumAIP and CumCRP and type 2 diabetes incidence stratified by dyslipidemia status

|  | | Combination of CumCRP and CumAIP, HRs (95% CIs) | | | | | | *P* value for interaction |
| --- | --- | --- | --- | --- | --- | --- | --- | --- |
|  | CumCRP<1 mg/L  CumAIP<-0.0701 | | 1≤CumCRP<3 mg/L  CumAIP<-0.0701 | CumCRP≥3 mg/L  CumAIP<-0.0701 | CumCRP<1 mg/L  CumAIP≥-0.0701 | 1≤CumCRP<3 mg/L  CumAIP≥-0.0701 | CumCRP≥3 mg/L  CumAIP≥-0.0701 |  |
| **Dyslipidemia ^*^** | | | | | | | | 0.0619 |
| Event/Total | 62/960 | | 177/1632 | 15/109 | 302/2201 | 1197/6591 | 66/328 |  |
| Incidence rate | 9.01 | | 15.72 | 20.14 | 20.07 | 26.71 | 29.54 |  |
| Unadjusted model | Reference | | 1.74 (1.31,2.33) | 2.25 (1.28,2.95) | 2.22 (1.69,2.92) | 2.96 (2.30,3.83) | 3.31 (2.34,4.67) |  |
| Model 1 | Reference | | 1.53 (1.14,2.04) | 1.74 (0.99,3.07) | 1.97 (1.50,2.60) | 2.32 (1.79,3.00) | 2.33 (1.64,3.32) |  |
| Model 2 | Reference | | 1.40 (1.05,1.88) | 1.61 (0.91,2.85) | 1.71 (1.29,2.25) | 1.97 (1.52,2.56) | 2.09 (1.27,2.98) |  |
| Model 3 | Reference | | 1.33 (1.00,1.79) | 1.58 (0.90,1.78) | 1.56 (1.18,2.06) | 1.70 (1.31,2.22) | 1.71 (1.19,2.44) |  |
| **Nondyslipidemia** | | | | | | | |  |
| Event/Total | 367/7126 | | 1026/11135 | 60/718 | 407/3724 | 1354/8341 | 85/495 |  |
| Incidence rate | 7.04 | | 12.86 | 11.51 | 15.50 | 23.77 | 24.52 |  |
| Unadjusted model | Reference | | 1.83 (1.63,2.06) | 1.68(1.28,2.21) | 1.19 (1.90,2.52) | 3.27 (3.01,3.79) | 3.57(2.82,4.52) |  |
| Model 1 | Reference | | 1.51 (1.34,1.71) | 1.25 (0.95,1.64) | 1.84 (1.60,2.13) | 2.39 (2.12,2.69) | 2.37 (1.87,3.00) |  |
| Model 2 | Reference | | 1.50 (1.33,1.69) | 1.40 (1.06,1.84) | 1.66 (1.44,1.92) | 2.21 (1.96,2.49) | 2.44 (1.92,3.10) |  |
| Model 3 | Reference | | 1.44 (1.28,1.63) | 1.33 (1.01,1.75) | 1.59 (1.38,1.83) | 1.97 (1.75,2.23) | 2.14 (1.68,2.72) |  |

Model 1: adjusted for age (continuous), education, smoking status, drinking status, physical exercise, family history of diabetes, BMI (continuous);

Model 2: Model 1+FBG (continuous), blood pressure (categorical), eGFR (categorical), antihypertensives (yes or no), lipid-lowering drugs (yes or no);

Model 3: model 2+ fatty liver (categorical).

^*^Dyslipidemia: TC≥6.2 mmol/L, TG≥2.25 mmol/L, LDL-C≥4.13 mmol/L, or HDL-C<1.03 mmol/L.

The incident rate is per 1,000 person-years.

Abbreviations: BMI, body mass index; CumAIP, cumulative atherogenic index of plasma; CumCRP, cumulative high-sensitivity C-reactive protein; eGFR, estimated glomerular filtration rate; FBG, fasting blood glucose; HDL-C, high-density lipoprotein cholesterol; HR, hazard ratio; LDL-C, low-density lipoprotein cholesterol; TC, total cholesterol; TG, triglyceride.

# Table S18. BasAIP-associated T2D in the entire cohort and across the baseline hsCRP thresholds (1, 3 mg/L)

|  | **BasAIP, HRs (95% CIs)** | | | | **Per SD** | ***P* for trend** |
| --- | --- | --- | --- | --- | --- | --- |
|  | **Quartile1** | **Quartile 2** | **Quartile 3** | **Quartile 4** |  |  |
| **Entire cohort** | | | | | | |
| Event/Total | 731/10841 | 1150/10848 | 1367/10831 | 1870/10840 |  |  |
| Incident rate | 9.3 | 15.01 | 18.01 | 25.33 |  |  |
| Model 1 | Reference | 1.43 (1.31,1.57) | 1.64 (1.50,1.81) | 2.09 (1.92,2.29) | 1.29 (1.26,1.33) | <0.001 |
| Model 2 | Reference | 1.34 (1.22,1.47) | 1.50 (1.36,1.64) | 1.78 (1.63,1.95) | 1.21 (1.18,1.24) | <0.001 |
| Model 3 | Reference | 1.30 (1.18,1.42) | 1.40 (1.28,1.54) | 1.54(1.40,1.69) | 1.14 (1.11,1.18) | <0.001 |
| **HsCRP<1 mg/L** | | | | | | |
| Event/Total | 305/5726 | 534/5561 | 617/5125 | 614/4075 |  |  |
| Incident rate | 7.23 | 13.38 | 16.86 | 21.88 |  |  |
| Model 1 | Reference | 1.59 (1.38,1.84) | 1.96 (1.71,2.26) | 2.26 (1.97,2.62) | 1.32 (1.26,1.38) | <0.001 |
| Model 2 | Reference | 1.52 (1.32,1.75) | 1.82 (1.58,2.09) | 2.01 (1.74,2.32) | 1.25 (1,20,1.31) | <0.001 |
| Model 3 | Reference | 1.47 (1.27,1.69) | 1.71 (1.49,1.97) | 1.74 (1.50,2.02) | 1.18 (1.13,1.24) | <0.001 |
| **1≤HsCRP<3 mg/L** | | | | | | |
| Event/Total | 267/3364 | 350/3290 | 416/3559 | 723/4089 |  |  |
| Incident rate | 11.14 | 15.12 | 16.77 | 25.89 |  |  |
| Model 1 | Reference | 1.26 (1.08,1.48) | 1.34 (1.14,1.56) | 1.99 (1.72,2.30) | 1.28 (1.23,1.34) | <0.001 |
| Model 2 | Reference | 1.17 (1.00,1.37) | 1.20 (1.02,1.40) | 1.63 (1.41,1.89) | 1.17 (1.12,1.23) | <0.001 |
| Model 3 | Reference | 1.13 (0.96,1.33) | 1.12 (0.96,1.31) | 1.44 (1.24,1.67) | 1.12 (1.07,1.17) | <0.001 |

**Table S18.** BasAIP-associated T2D in the entire cohort and across the baseline hsCRP thresholds (1, 3 mg/L) (continued)

|  | **BasAIP, HRs (95% CIs)** | | | | **Per SD** | ***P* for trend** |
| --- | --- | --- | --- | --- | --- | --- |
|  | **Quartile1** | **Quartile 2** | **Quartile 3** | **Quartile 4** |  |  |
| **HsCRP≥3 mg/L** | | | | | | |
| Event/Total | 159/1751 | 266/1997 | 334/2147 | 533/2676 |  |  |
| Incident rate | 12.89 | 19.64 | 23.02 | 29.89 |  |  |
| Model 1 | Reference | 1.37 (1.13,1.67) | 1.55 (1.28,1.88) | 1.83 (1.52,2.20) | 1.26 (1.19,1.33) | <0.001 |
| Model 2 | Reference | 1.28 (1.05,1.56) | 1.45 (1.20,1.76) | 1.58 (1.31,1.90) | 1.18 (1.12,1.25) | <0.001 |
| Model 3 | Reference | 1.25 (1.03,1.53) | 1.38 (1.14,1.68) | 1.37(1.14,1.66) | 1.11 (1.05,1.18) | <0.001 |
| *P* for INTm: BasAIP quartile × BasCRP thresholds (1, 3 mg/L) =0.0011; BasAIP quartiles × logBasCRP<0.001. | | | | | | |

Quartile 1: BasAIP<-0.2605; Quartile 2: -0.2605≤BasAIP<-0.0734; Quartile 3: -0.0734≤BasAIP<0.1257; Quartile 4: BasAIP≥0.1257.

Model 1: adjusted for age, sex, education, smoking status, drinking status, physical activities, family history of diabetes, and BMI (continuous);

Model 2: model 1 + FBG (continuous), TC (continuous), blood pressure (categorical), eGFR (categorical), antihypertensives (yes or no), lipid-lowering drugs (yes or no), logBasCRP (limited to the entire cohort);

Model 3: model 2+ fatty liver (categorical).

The incidence rate is per 1,000 person-years.

Per SD: risk per SD increase in BasAIP (0.3095).

Abbreviations: BasAIP, baseline atherogenic index of plasma; BasCRP, baseline high-sensitivity C-reactive protein; BMI, body mass index; eGFR, estimated glomerular filtration rate; FBG, fasting blood glucose; HRs, hazard ratios; INTm, multiplicative interact; TC, total cholesterol.

# Table S19. Type 2 diabetes risk on co-exposure stratified by BasCRP thresholds (1, 3 mg/L) and BasAIP (median)

|  | **Combination of BasCRP and BasAIP, HRs (95% CIs)** | | | | | |
| --- | --- | --- | --- | --- | --- | --- |
|  | **BasCRP<1 mg/L**  & **BasAIP<-0.0734** | **1≤BasCRP<3 mg/L** & **BasAIP<-0.0734** | **BasCRP≥3 mg/L** & **BasAIP<-0.0734** | **BasCRP<1 mg/L** & **BasAIP≥-0.0734** | **1≤BasCRP<3 mg/L** & **BasAIP≥-0.0734** | **BasCRP≥3 mg/L** & **BasAIP≥-0.0734** |
| Event/Total | 839/11287 | 617/6654 | 425/3748 | 1231/9200 | 1139/7648 | 867/4823 |
| Incidence rate | 10.223 | 13.10 | 16.12 | 19.04 | 21.60 | 26.81 |
| Unadjusted model | Reference | 1.28 (1.16,1.42) | 1.60 (1.42,1.79) | 1.85 (1.70,2.02) | 2.11 (1.93,2.30) | 2.61 (2.38,2.87) |
| Model 1 | Reference | 1.09 (0.98,1.21) | 1.29 (1.15,1.45) | 1.61 (1.47,1.76) | 1.57 (1.43,1.72) | 1.82 (1.65,2.01) |
| Model 2 | Reference | 1.08 (0.97,1.20) | 1.21 (1.07,1.36) | 1.46 (1.33,1.60) | 1.39 (1.27,1.52) | 1.61 (1.46,1.78) |
| Model 3 | Reference | 1.03 (0.93,1.14) | 1.13 (1.01,1.27) | 1.37 (1.25,1.50) | 1.21 (1.10,1.33) | 1.35 (1.22,1.50) |
|  | **1≤BasCRP<3 mg/L & BasAIP<-0.0734** | **BasCRP<1 mg/L & BasAIP<-0.0734** | **BasCRP≥3 mg/L & BasAIP<-0.0734** | **BasCRP<1 mg/L & BasAIP≥-0.0734** | **1≤BasCRP<3 mg/L & BasAIP≥-0.0734** | **BasCRP≥3 mg/L & BasAIP≥-0.0734** |
| Model 1 | Reference | 0.92(0.83,1.02) | 1.18(1.04,1.34) | 1.47(1.34,1.62) | 1.44(1.30,1.62) | 1.67(1.50,1.85) |
| Model 2 | Reference | 0.93 (0.84,1.03) | 1.12 (0.99,1.27) | 1.35 (1.23,1.49) | 1.29 (1.17,1.42) | 1.49 (1.35,1.66) |
| Model 3 | Reference | 0.97 (0.88,1.08) | 1.10 (0.97,1.25) | 1.33 (1.21,1.47) | 1.18 (1.07,1.30) | 1.32 (1.18,1.46) |
| *P*-INTm: BasCRP (1, 3 mg/L) × BasAIP (median) = 0.0523. | | | | | | |

Model 1: adjusted for age, sex, education, smoking status, drinking status, physical activities, family history of diabetes, and BMI (continuous);

Model 2: model 1 + FBG (continuous), TC (continuous), blood pressure (categorical), eGFR (categorical), antihypertensives (yes or no), lipid-lowering drugs (yes or no);

Model 3: model 2+ fatty liver (categorical).

The incidence rate is per 1,000 person-years.

Abbreviations: BasAIP, baseline atherogenic index of plasma; BasCRP, baseline high-sensitivity C-reactive protein; BMI, body mass index; eGFR, estimated glomerular filtration rate; FBG, fasting blood glucose; HRs, hazard ratios; INTm, multiplicative interact; TC, total cholesterol.


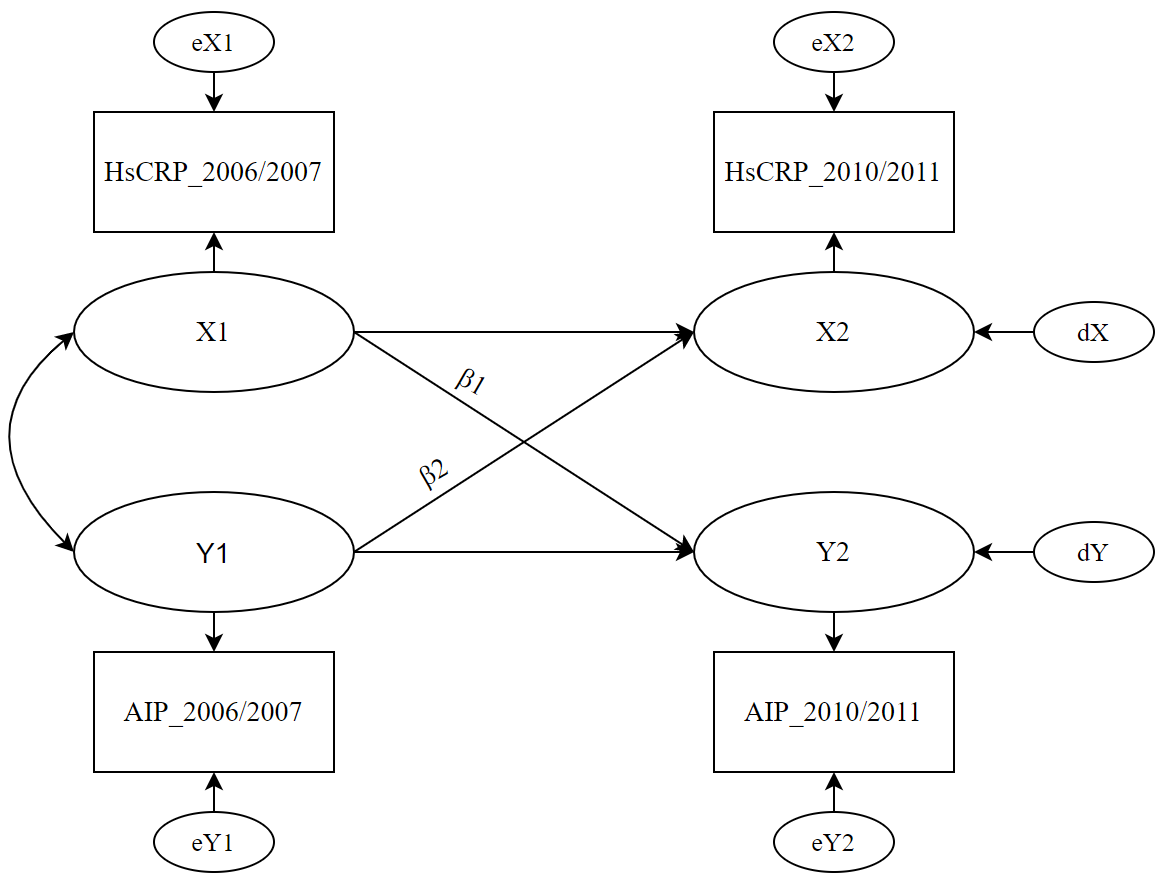


# Fig. S1. Cross-lagged analysis design panel

This design measured the effect size of hsCRP measured at 2006/2007 on subsequent AIP measured at 2010/2011 (β1) and the effect size of AIP measured at 2006/2007 on subsequent hsCRP measured at 2010/2011 (β2) simultaneously, adjusting for the auto-regressive

effects. Abbreviations: hsCRP: high-sensitivity C-reactive protein; AIP: atherogenic index of plasma.
